# Neurotherapeutic impact of vanillic acid and ibudilast on the cuprizone model of multiple sclerosis

**DOI:** 10.3389/fnmol.2024.1503396

**Published:** 2025-01-10

**Authors:** Rasha M. Alderbi, Mohammad Z. Alam, Badrah S. Alghamdi, Hadeil M. Alsufiani, Gamal S. Abd El-Aziz, Ulfat M. Omar, Maryam A. Al-Ghamdi

**Affiliations:** ^1^Research Centre, King Faisal Specialist Hospital and Research Centre, Jeddah, Saudi Arabia; ^2^Biochemistry Department, Faculty of Science, King Abdulaziz University, Jeddah, Saudi Arabia; ^3^Neuroscience and Geroscience Research Unit, King Fahd Medical Research Center, King Abdulaziz University, Jeddah, Saudi Arabia; ^4^Department of Medical Laboratory Sciences, Faculty of Applied Medical Sciences, King Abdulaziz University, Jeddah, Saudi Arabia; ^5^Department of Physiology, Neuroscience Unit, Faculty of Medicine, King Abdulaziz University, Jeddah, Saudi Arabia; ^6^Department of Clinical Anatomy, Faculty of Medicine, King Abdulaziz University, Jeddah, Saudi Arabia; ^7^Princess Dr. Najlaa Bint Saud Al-Saud Center of Excellence Research in Biotechnology, King Abdulaziz University, Jeddah, Saudi Arabia; ^8^Vitamin D Pharmacogenomics Research Group, King Abdulaziz University, Jeddah, Saudi Arabia; ^9^Experimental Biochemistry Unit, King Fahd Medical Research Center, King Abdulaziz University, Jeddah, Saudi Arabia

**Keywords:** multiple sclerosis, cuprizone model, vanillic acid, ibudilast, anti-inflammatory

## Abstract

Multiple sclerosis (MS) affects 2.8 million people worldwide. Although the cause is unknown, various risk factors might be involved. MS involves the immune system attacking the central nervous system’s myelin sheath, leading to neuron damage. This study used a cuprizone (CPZ)-intoxicated mouse model to simulate MS’s demyelination/remyelination process. It evaluated the molecular, histological, and behavioral effects of vanillic acid (VA), a natural phenolic acid, alone and with Ibudilast (IBD), a clinically tested MS medication. Mice were divided into a control group (regular chow) and a CPZ group (0.3% cuprizone chow for 5 consecutive weeks). During remyelination, the CPZ group was split into four groups: no therapy, 10 mg/kg of IBD, 30 mg/kg of VA, and combined, each treated for 4 weeks. Behavioral, biochemical, molecular, and histopathological tests occurred in the 5^th^ week (demyelination), 7^th^ (early remyelination), and 9^th^ (late remyelination). Cognitive assessments were at weeks 5 and 9. VA enhanced motor, coordination, and cognitive impairments in CPZ-intoxicated mice and improved histopathological, molecular, and biochemical features during early remyelination. IBD improved behavioral abnormalities across all tests, but combined therapy showed no significant difference from single therapies. Further investigations are necessary to understand VA’s mechanisms and potential as an MS treatment.

## 1 Introduction

Multiple sclerosis (MS) is a chronic, neurodegenerative autoimmune disease targeting the myelin sheath of primarily the central nervous system (CNS). With an estimated 2.8 million cases in 2020, the global prevalence of MS increased from 29.26 to 43.95 per 100,000 population compared with 2013. Notably, at least twice as many females as males are at risk of developing MS (Walton et al., 2020). Common symptoms include optic neuritis, myelitis, motor deficits, imbalance, sensory and cognitive impairments, fatigue, heat sensitivity, bladder and bowel dysfunction, headache, and depression. These symptoms vary depending on the affected area and disease stage (Gelfand, 2014). There is no single diagnostic criterion for MS; however, diagnosis can involve identifying symptoms, magnetic resonance imaging (MRI) showing CNS lesions, cerebrospinal fluid analysis, and evoked potential tests (Milo and Miller, 2014). While the exact origin remains uncertain, several factors are associated with MS development. These factors includes genetic predisposition, air pollution, vitamin D deficiency, Epstein–Barr virus exposure, aging, obesity, smoking, physical activity, psychological stress, gender, hormones, inflammation, and the microbiome (Boziki et al., 2020; Hecker et al., 2021).

MS targets the myelinated axons in the CNS, resulting in the destruction of the myelin and axons to different extents. The demyelination of white and gray matter is due to the interactions between brain cells (microglia and astrocytes) and peripheral immune cells (lymphocytes and monocytes). In addition to demyelination, immune cell infiltration and activation may contribute to the destruction of nerve cells. This process plays a role in the development of cerebral atrophy and axonal damage, both of which are hallmarks of the advanced phases of the disease (Dobson and Giovannoni, 2019).

It has been proposed that the inflammatory process associated with MS is initiated by one or more of the previously mentioned risk factors. This results in the disruption of the blood–brain barrier (BBB) through the activation of myeline-reactive T cells in the periphery. Consequently, leukocytes from the bloodstream transmigrate to the CNS through the disrupted BBB. Antigen-presenting cells, including dendritic cells, microglia, B cells, and macrophages, then reactivate T cells, eliciting an inflammatory response. This inflammation leads to the recruitment of inflammatory cells (monocytes, T cells, and B cells), the release of cytokines and chemokines, and the activation of microglia and macrophages. Together, these factors cause lesion formation and demyelination of oligodendrocytes, resulting in neurological dysfunctions (Talanki Manjunatha et al., 2022).

Remyelination that occurs spontaneously after demyelination is frequently incomplete. Thus, identifying the causes of remyelination failure and devising techniques to restore myelin constitute the obstacles in MS research (Han et al., 2020).

The cuprizone (CPZ) mouse model of demyelination serves as a highly effective model for investigating the pathogenesis and treatment of MS. CPZ (bis-cyclohexanone oxaldihydrazone) is a copper-chelating agent administered to rodents, resulting in demyelination that resembles MS in humans. Some of the symptoms that have been noticed in people with MS including motor-related symptoms, such as tiredness, weakness, loss of vision, loss of balance, loss of speech, muscular spasms, digestive and urinary incontinence, and even seizures, were observed in the CPZ model of demyelination (Herring and Konradi, 2011). Cognitive impairments, such as memory loss, anxiety, and social deficits, have also been found in the CPZ model like MS patients. The demyelination of the corpus callosum (CC) in the CPZ model has been associated with decreased motor coordination between the left and right paws mimicking the imbalance seen in MS patients (Liebetanz and Merkler, 2006). Further, there is a correlation between the severity of hippocampal lesions in MS and cognitive failure, particularly in long-term episodic memory. Substantial demyelination in the hippocampus has been reported following CPZ intoxication (Norkute et al., 2009). To induce acute demyelination, mice are administered CPZ either orally or mixed with rodent chow for 5–6 consecutive weeks. Subsequently, CPZ is withheld for 4 weeks to facilitate spontaneous remyelination. For chronic demyelination, a prolonged period of demyelination induction can be implemented. During the demyelination period, mice demonstrate disrupted behaviors, motor deficits, and molecular alterations similar to those observed in MS patients (Praet et al., 2014; Torkildsen et al., 2008; Zirngibl et al., 2022; Supplementary Figure S1).

As MS has become more prevalent worldwide, there has been an increase in necessity for inventing a medication that treats MS (AlJumah et al., 2020; Walton et al., 2020). To date, all the therapies available are either aimed at managing the symptoms of MS or at limiting the activity of the disorder during the relapsing phase. However, the progressive phase is not being addressed by any of these medicines (Okuda et al., 2009).

Vanillic acid (VA) is a phenolic acid with a pale-yellow hue and a creamy scent, found in many plants such as vanilla beans (Ranadive, 1992), pumpkin seeds (Mitić et al., 2020), papaya, mango, banana (Siriamornpun and Kaewseejan, 2017), and various berries (Mattila et al., 2006). VA possesses anti-cancer, anti-fungal, antidepressant, antinociceptive, antioxidant, anti-inflammatory and neuroprotective activities (Ingole et al., 2021). Due to their potent antioxidant and anti-inflammatory properties, phenols like VA have been found to benefit neurological disorders (Babazadeh et al., 2023; Sharma et al., 2020). Nonetheless, little is known regarding the underlying molecular pathways responsible for VA’s neuroprotective benefits.

Ibudilast (IBD) is an organic compound classified as a pyrazolopyridine and has been utilized in Asia for the past 2 decades. Initially employed to manage bronchial asthma, it has recently been found effective in treating post-stroke dizziness and ocular allergies, with a commendable safety profile for patients (Cui et al., 2014). IBD exhibits various biological actions, such as anti-inflammation effects and neuro-immune modulation (Goodman et al., 2016; Grodin et al., 2021). It acts as a phosphodiesterase (PDE) inhibitor with non-selective inhibitory effects on PDE3, PDE4, PDE10, and PDE11 (Cui et al., 2014). A clinical trial was conducted to explore the potential of IBD as a treatment for MS. In phase II of the trial, IBD did not show improvement in acute MS relapses but demonstrated enhanced effects on brain atrophy in patients with secondary progressive MS (SPMS) and primary progressive MS (PPMS) (Fox et al., 2018). Consequently, the U.S. Food and Drug Administration has approved phase III trials, with Medicinova Inc. sponsoring further evaluation of IBD in SPMS and PPMS patients (O’Brien, 2019).

The growing incidence of MS worldwide has increased the need for effective treatments and potential prevention strategies. Currently, existing therapies primarily manage symptoms or reduce disease manifestations during relapses, without addressing the progressive phase. Thus, this research aimed to examine the therapeutic efficacy of the natural product VA and the anti-inflammatory medication IBD, both individually and in combination, using CPZ-induced mouse model of MS.

## 2 Materials and methods

### 2.1 Animals

A total of 88 male SWR/J mice, aged 6–8 weeks and weighing 15–22 g, were purchased from the Animal House of King Fahd Medical Research Center, Jeddah, Saudi Arabia. All experiments adhered to the guidelines of the Animal Care and Use Committee (ACUC) (ACUC-20-10-24), Research Ethics Committee of Unit of Biomedical Ethics (Reference No 617–20) at the Faculty of Medicine, King Abdulaziz University, Jeddah, Saudi Arabia, and the Institutional Review Board (IRB) at King Faisal Specialist Hospital and Research Center (RAC No. 2024–72), Jeddah, Saudi Arabia. The mice were housed under standard laboratory conditions with a 12-h light/dark cycle at 22°C ± 3°C, with water and food provided ad libitum.

### 2.2 Chemicals and drugs

#### 2.2.1 Cuprizone

CPZ, oxalic acid bis (cyclohexylidenehydrazide) (CAT. # 1018410025), was purchased from Sigma-Aldrich, St. Louis, MO, United States. For 0.3% CPZ chaw preparation, standard rodent chow was grounded and mixed properly with CPZ to achieve a 0.3% concentration (w/w) (Zhan et al., 2020).

#### 2.2.2 Dimethylsulfoxide

Dimethylsulfoxide (DMSO) (CAT. # 31089) was purchased from Techno Pharmchem, New Delhi, India, and used as a solvent for all treatments. A 5% DMSO solution in normal saline (0.9% w/v sodium chloride) freshly prepared daily for use in the treatments.

#### 2.2.3 Ibudilast

IBD, (CAT. # A11019), was purchased from Adooq Bioscience, Irvine, CA, USA. For accurate dosing, a concentration of 10 𝜇g/g was prepared for each mouse based on weekly weight, achieving a dosage of 10 mg/kg for intraperitoneal (I.P.) injection. The IBD was dissolved in 5% DMSO (Wu and Wang, 2020).

#### 2.2.4 Vanillic acid

VA (CAT. # MFCD00002551) was purchased from Sigma-Aldrich, St. Louis, MO, United States. For a dosage of 30 mg/kg, a concentration of 30 𝜇g/g was calculated for each mouse according to its weekly weight and dissolved in 5% DMSO (Ullah et al., 2020). The total volume of daily injections was kept within a maximum of 20 IU/mouse (Andrews, 2014).

### 2.3 Experimental design

The study lasted 9 weeks in total, with 5 weeks dedicated to the demyelination phase and the remaining 4 weeks representing spontaneous remyelination. Week 7, the middle time point of the remyelination phase, was crucial for assessing early remyelination. Mice weights were measured weekly to monitor changes due to CPZ or treatments, and behavioral tests were conducted at specific intervals: weeks 5, 7, and 9 (Supplementary Figures S2, S3). At the end of the demyelination phase in week 5, some mice from each group were sacrificed for histopathological, biochemical, and molecular analyses to confirm disease modeling. Half of the remaining mice from each group were sacrificed in week 7 for similar analyses, and the rest were sacrificed at the end of the study in week 9 for the same types of analyses. Immediate head dissection was performed using sharp, small tools to extract the brain on a cold surface while maintaining tissue integrity. The collected brains were sectioned sagittally and divided into three groups: the first group was preserved in RNAlater® solution (Thermofisher Scientific, CAT. # AM7024) for biomolecular analyses, the second was fixed and embedded in paraffin blocks for histopathology and immunohistochemistry (IHC), and the third was preserved at −80°C for oxidative stress biomarker assays.

### 2.4 Grouping

SWR/J male mice were divided into two main groups: the control group (20%) and the CPZ group (80% mice). To induce demyelination, the CPZ group was fed 0.3% mixed rodent chow for 5 consecutive weeks, whereas the control group received normal rodent chow during the same period. Following this, the remaining mice in the CPZ group were further subdivided into four equal groups (20% mice/ each): the CPZ (disease model) group, the IBD-treated group, the VA-treated group, and the VA + IBD combination-treated group. The control group was also included during the treatment phase (Supplementary Figure S4).

### 2.5 Behavioral tests

#### 2.5.1 Locomotion tests

##### 2.5.1.1 Wire hang test

Briefly, each mouse was placed on a horizontal wire and encouraged to grasp it while latency to fall time was recorded not exceeding 60 s (sec.). Three trials were performed for each mouse and the average time was calculated in seconds. (Au - Aartsma-Rus and Au - van Putten, 2014).

##### 2.5.1.2 Grip strength test

To assess neuromuscular function in rodents, the GS test was performed. This test determines the maximum peak force exerted by a rodent as the experimenter attempts to pull it from a specially designed grid. Forelimb strength (g force) was measured using a GS meter (Columbus Instruments, Columbus, OH, United States). Mice were held by the tail and allowed to grip the grid with their forelimbs. Once a firm grip was achieved, the mice were pulled away until their grasp was broken, and the grip force was recorded. Each mouse underwent three trials, and the mean values (g) were recorded and normalized to the body weight (g/body weight) of each mouse.

##### 2.5.1.3 Open-field test

To evaluate the effects of CPZ and melatonin on motor behavior, we conducted the OFT weekly, using an established protocol widely applied to evaluate motor function in rodents (Balkaya et al., 2013). Each mouse was placed in an open-field arena (45 cm × 45 cm square) at the beginning of the test and allowed to move freely for 3 min. The movement of the mice was tracked and recorded by a video camera positioned above the box. The EthoVision tracking system was used to measure the TDM (cm) and velocity (cm/s).

#### 2.5.2 Coordination and balance test

##### 2.5.2.1 Rotarod test

The apparatus of the RR test consists of a rotating cylinder (rod) which rotates with an accelerating speed from 4 to 40 revolutions per minute (rpm) while mice were encouraged to move and keep balanced. Each mouse was given 3 trials to accomplish 300 s of the test. Latency to fall in sec. and rotating speed in rpm were recorded then average of the trials was calculated (Karl et al., 2003).

#### 2.5.3 Cognition tests

##### 2.5.3.1 Y-maze spontaneous alternation test

The Y-maze spontaneous alternation task is commonly used to evaluate short-term spatial working memory in mice. The test was performed as described earlier (Aldhahri et al., 2022). Mice were placed in a Y-maze apparatus (10-cm width and 40-cm height) with three identical arms separated equally by 120° angles. Each mouse was positioned at the end of one arm facing the wall and allowed to explore the maze freely for 8 min. When a mouse visited three arms consecutively an alteration was counted. Arm entries and alternations between the arms were recorded to calculate the percentage of spontaneous alteration using the following equation: % alternation = [number of alternations/total arm visit −2] × 100.

To assess short-term spatial memory in mice, the Y-maze spontaneous alternation test is frequently employed. The procedure for conducting the Y-maze spontaneous alternation test was as described by Aldhahri et al. (2022). The Y-shaped maze comprises three arms set at a 120° angle, each arm measuring 40 cm in length and10 cm in width. The mice were placed at the end of one arm facing the wall and allowed to explore the maze freely for 8 min. The proportion of spontaneous alteration was determined by recording arm entries and alternations and applying the following formula:

% alternation = [number of alternations/total arm visit −2] × 100 (Shi et al., 2021).

##### 2.5.3.2 Novel object recognition test

To assess the impact of CPZ and other therapies on short-term recognition memory, we performed the NORT using the same arena and recording system of the OFT. The methodology previously reported by our laboratory was followed (Aldhahri et al., 2022; Lababan, 2021). The NORT comprises two stages: the familiarization phase and the test phase. Prior to the familiarization phase, the mice underwent a habituation experiment, where each mouse was given the opportunity to explore the empty arena for 3 min. After a 24-h period, the mice underwent the familiarization phase, where each mouse was given the opportunity to investigate two identical objects (referred to as familiar 1 and familiar 2) placed in the same arena. The exploration time for each mouse was limited to 3 min. The test phase was conducted 10 min after the familiarization phase was completed. During the test phase, one of the familiar objects was substituted with a novel object that differed in shape and color. Subsequently, the mice were given 3 min to investigate the objects. The experiment was conducted in a quiet environment, and the selected objects (familiar and novel) were cleanable and of substantial weight. Following each trial, the arena and objects were thoroughly sanitized using a 10% ethanol solution to eliminate scent cues. Normal mice tend to spend longer time exploring the novel object compared with the familiar one. The parameters used for interpretation were the DI and the PI, calculated as follows:

$$DI=\frac{\mathrm{Novel}\;\mathrm{object}\;\mathrm{exploration}\;\mathrm{time}-\mathrm{Familiar}\;\mathrm{object}\;\mathrm{exploration}\;\mathrm{time}\;}{\mathrm{Total}\;\mathrm{objects}\;\mathrm{exploration}\;\mathrm{time}}$$

$$\mathrm{PI}=\frac{\mathrm{Novel}\;\mathrm{object}\;\mathrm{exploration}\;\mathrm{time}}{\mathrm{Total}\;\mathrm{objects}\;\mathrm{exploration}\;\mathrm{time}}\times 100$$

The EthoVision tracking system (XT8A system, Noldus Information Technology, Wageningen, The Netherlands) was used to record all the parameters. The DI values range from 0 (no exploration of the new object) to 1 (exclusive exploration of the new object). The PI can range from 0% (no exploration of the new object) to 100% (exclusive exploration of the new object). A value of 50% indicates that equal time is spent examining the novel and the familiar objects.

### 2.6 Gene expression

RNA extraction was conducted using TRIzol™ Reagent (CAT. # 15596026) purchased from Thermo Fisher Scientific. First, the brains in RNAlater® were removed from storage at −80°C and allowed to thaw at ambient temperature for 1 h. Subsequently, 100 mg of brain tissue was used for RNA extraction following the manufacturer’s protocol. The RNA was then reverse transcribed to cDNA using the SuperScript™ IV First-Strand Synthesis System Kit (CAT. #: 18091050), as per the manufacturer’s instructions. The expression of the targeted genes, interleukin-4 (Il-4) and cyclooxygenase-2 (Cox-2), was evaluated using quantitative reverse transcription polymerase chain reaction (RT-qPCR). The housekeeping gene used for comparison was glyceraldehyde 3-phosphate dehydrogenase (Gapdh). In summary, 100 ng of the produced cDNA was combined with 5 μL of PowerUp™ SYBR™ Green PCR Master Mix (CAT. # A25741, Thermo Fisher Scientific), 10 pmol of forward and reverse primers (listed in Table 1), and nuclease-free water to make a total volume of 10 μL. The PCR conditions were set according to the standard cycling mode outlined in the kit manual. This involved the following steps: (1) Activation of Uracil-DNA glycosylase at 50°C for 2 min (performed once). (2) Activation of Dual-Lock™ DNA polymerase at 95°C for 2 min (performed once). (3) A total of 40 cycles were carried out, consisting of denaturation at 95°C for 15 s, followed by annealing and extension at 60°C for 1 min (if the primer’s melting temperature was equal to or greater than 60°C). The annealing temperature for primer sets with a Tm below 60°C was calculated individually within the range of 52°C–59°C, followed by extension at 72°C. The qPCR tests were conducted using the StepOne™ Real-Time PCR System from Applied Biosystems. The relative fold gene expression of the samples was computed using the delta–delta Ct method.

**Table 1:** Primers Sequences used in RT-qPCR.

Gene Symbol	Forward Primer (5` → 3`)	Reverse Primer (5` → 3`)
Gapdh	AGGTCGGTGTGAACGGATTTG	GGGGTCGTTGATGGCAACA
Il-4	GGTCTCAACCCCCAGCTAGT	GCCGATGATCTCTCTCAAGTGAT
Cox-2	TGAGTACCGCAAACGCTTCT	CAGCCATTTCCTTCTCTCCTGT

### 2.7 Cholinergic enzyme activity

#### 2.7.1 Acetylcholinesterase activity assay

The AChE Activity Assay Kit (CAT. # BC2020) was purchased from Solarbio Science and Technology Co., Ltd., Beijing, China. The assay was performed according to the manufacturer’s manual.

### 2.8 Histopathology and immunohistochemistry

Paraffin blocks were sectioned into 4𝜇m-thick slices and placed on positively charged slides for staining. The LFB Myelin Stain Kit (Cresyl Violet Method), CAT. # G3245 was purchased from Solarbio Science & Technology Co., Ltd., Beijing, China, and slides were stained according to the manufacturer’s protocol. MBP Polyclonal Antibody, CAT. # MBS175140, was purchased from MyBioSource to assess the myelin content in mice brains using IHC using automatic staining machine, the Ventana BenchMark GX. For slide imaging, an Olympus DP72 camera attached to an Olympus BX51 light microscope was used with CellSens Standard software.

### 2.9 Statistical analyses

GraphPad Prism 10 (San Diego, CA, United States) software was used for statistical analyses. For tests and analyses performed at the demyelination time point (week 5), an unpaired two-tailed *t* test was used (with Welch’s correction where applicable), while one-way ANOVA followed by the Tukey multiple comparison test was used for the remaining time points (weeks 7 and 9) (with Welch and Brown–Forsythe’s corrections where applicable). Data were presented as mean ± standard error of mean. Differences between groups were considered statistically significant at *p* < 0.05.

## 3 Results

### 3.1 Behavioral tests

#### 3.1.1 Locomotion tests

##### 3.1.1.1 Wire hang test

**Figure 1:**
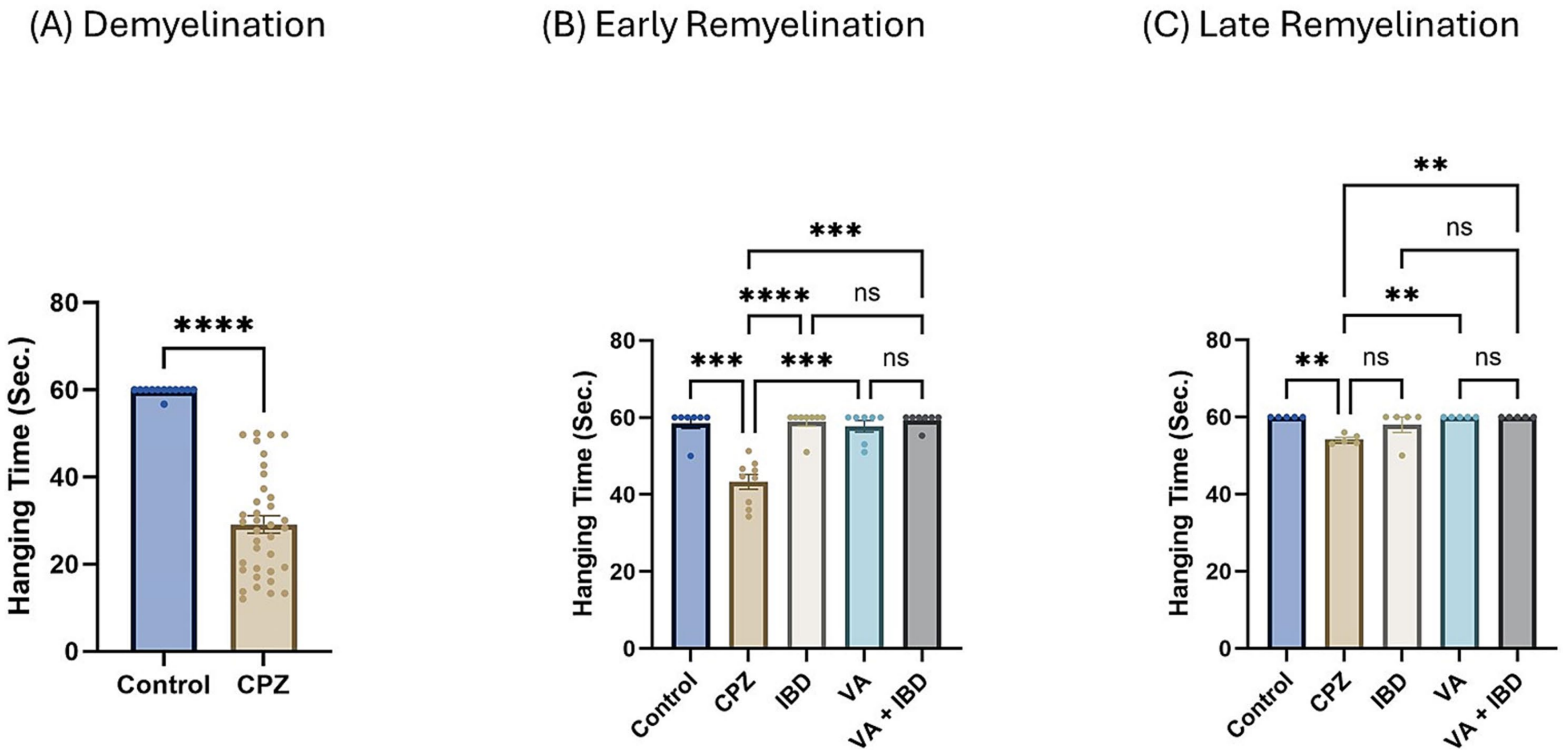
Wire Hang test. **(A)** Hanging time in week 5 of demyelination in control compared to CPZ group. **(B)** Hanging time in early remyelination at week 7 was shown in control and treated groups (IBD, VA, VA + IBD) compared to CPZ. **(C)** Hanging time in late remyelination at week 9 was displayed for control, CPZ, IBD, VA, and VA + IBD group. Data in **(A)** was analyzed by unpaired two-tailed *t* test. Data in **(B)** and **(C)** was analyzed using one-way ANOVA followed by Tukey’s multiple comparisons test. Data was presented as mean ± SEM, and *p* < 0.05 was considered significantly different. ns, non-significant, *p < 0.05, **p < 0.01, ***p < 0.001, and ****p < 0.0001. Sec., seconds.

At the end of the demyelination phase, a significant decrease in hanging time was observed in the CPZ group compared with the control group (Figure 1A). In the early remyelination stage (week 7), all the treated groups (IBD, VA, and VA + IBD) have shown significant enhancement in hanging time compared with the CPZ group, which was not given any treatment after week 5 and left to remyelinate spontaneously. However, no significant difference in hanging time was noted between combined and individual treatments (Figure 1B). In the late remyelination stage at week 9, the VA and VA + IBD groups, as well as the control group, exhibited a significant increase in hanging time compared with the CPZ group, whereas IBD alone exhibited no significant difference. Moreover, no significant difference was observed in the VA + IBD group compared with the VA and IBD groups (Figure 1C).

##### 3.1.1.2 Grip strength test

**Figure 2:**
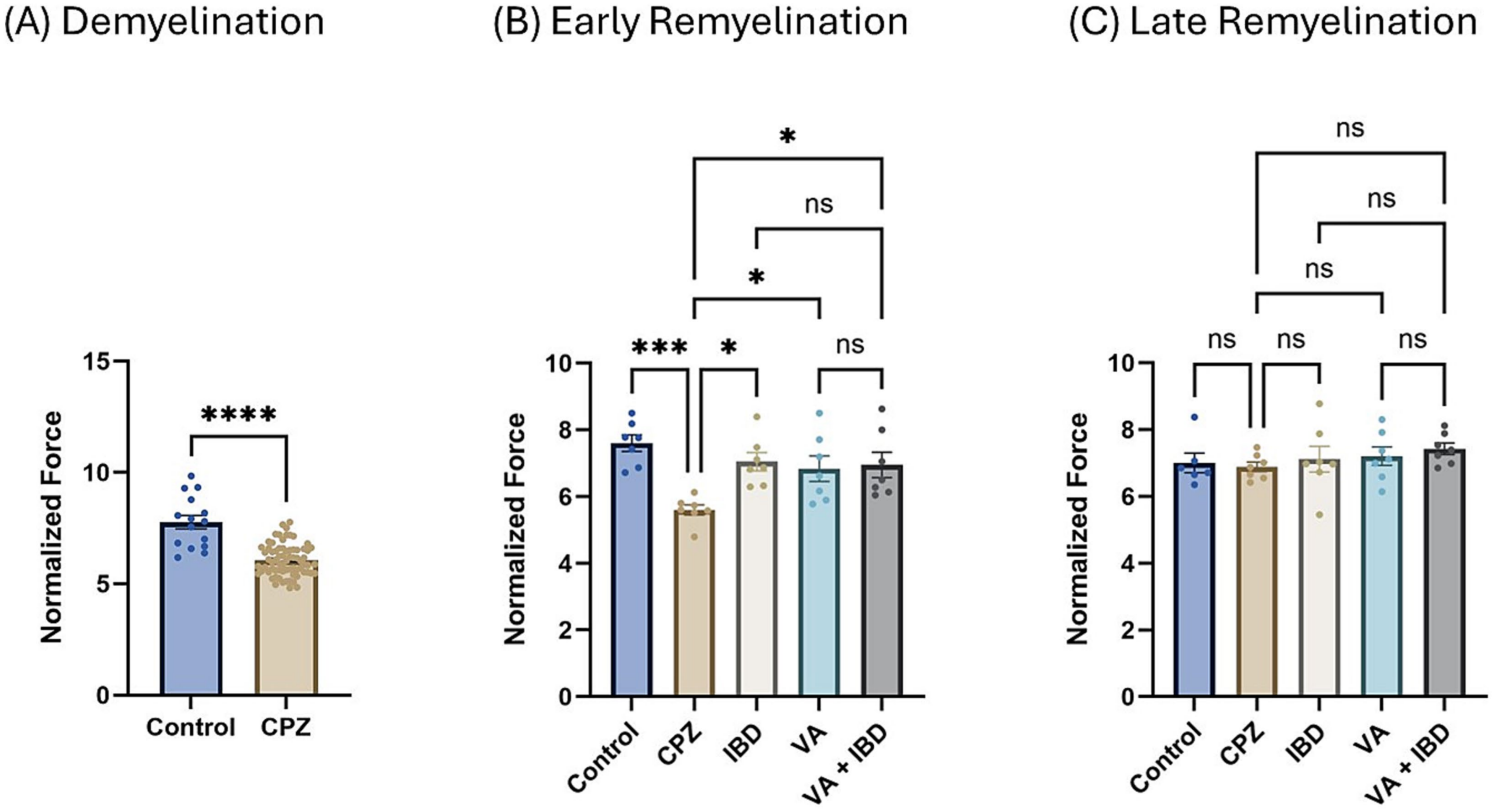
Grip strength (GS) expressed as normalized force. **(A)** GS measured in week 5 of demyelination represented by unpaired two-tailed t test. **(B)** GS in early remyelination measured in week 7. **(C)** GS in late remyelination measured in week 9. Both **(B,C)** were analyzed using one-way ANOVA followed by Tukey’s multiple comparison test. Data is presented as mean ± SEM. *p* < 0.05 was considered significantly different. ns, non-significant, *p < 0.05; **p < 0.01; ***p < 0.001; and ****p < 0.0001.

Normalized force for all forelimbs and hindlimbs was measured using a grip strength (GS) meter. At the end of the demyelination stage, a significant decrease in GS was observed in the CPZ group compared with the control group (Figure 2A). In the early remyelination stage (week 7), the IBD, VA, and combined treatment groups, along with the control group, exhibited a significant increase in GS compared with the CPZ group, which underwent spontaneous remyelination. However, no significant difference in GS was observed between the combined and individual treatments (Figure 2B). At the end of the late remyelination stage (week 9), no significant difference in GS was observed between the CPZ group and any of the other groups, suggesting that remyelination was likely complete (Figure 2C).

##### 3.1.1.3 Open-field test

**Figure 3:**
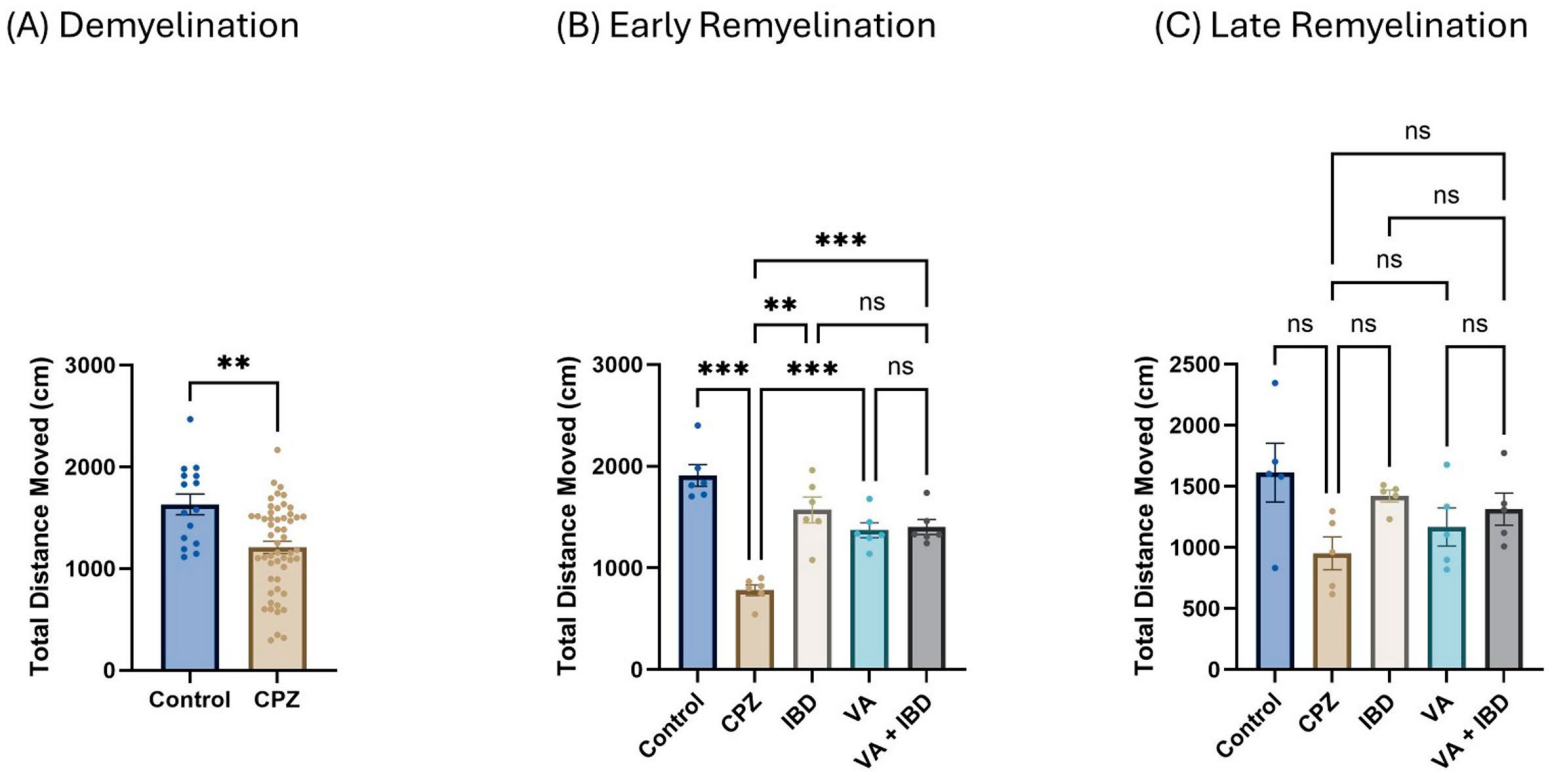
The total distance moved (TDM) in open field test (OFT). **(A)** Displayed week 5 TDM during demyelination. **(B)** Showed TDM in early remyelination at week 7 in control, CPZ, and treated groups. **(C)** Showed TDM in late remyelination in week 9. Data in **(A)** was analyzed by unpaired two-tailed t test. Data in **(B,C)** was analyzed using one-way ANOVA followed by Tukey’s multiple comparisons test. Data was presented as mean ± SEM, and p < 0.05 was considered significantly different. ns, non-significant; *p < 0.05; **p < 0.01; and ***p < 0.001. OFT, open field test; cm, centimeter.

The total distance moved (TDM) in centimeters (cm) was measured during the open-field test (OFT). In the CPZ group, a significant decrease in the TDM was noted in contrast with the control group in week 5 of demyelination (Figure 3A). Notably, a significant improvement was observed in the IBD, VA, and VA + IBD groups compared with the control group in terms of the TDM. No significant difference was observed between the treatment groups following 2 weeks of treatment (early remyelination) (Figure 3B). At the end of the late remyelination stage, no difference in the TDM was found between the CPZ group and all other remaining groups (Figure 3C).

#### 3.1.2 Coordination and balance test

##### 3.1.2.1 Rotarod test

**Figure 4:**
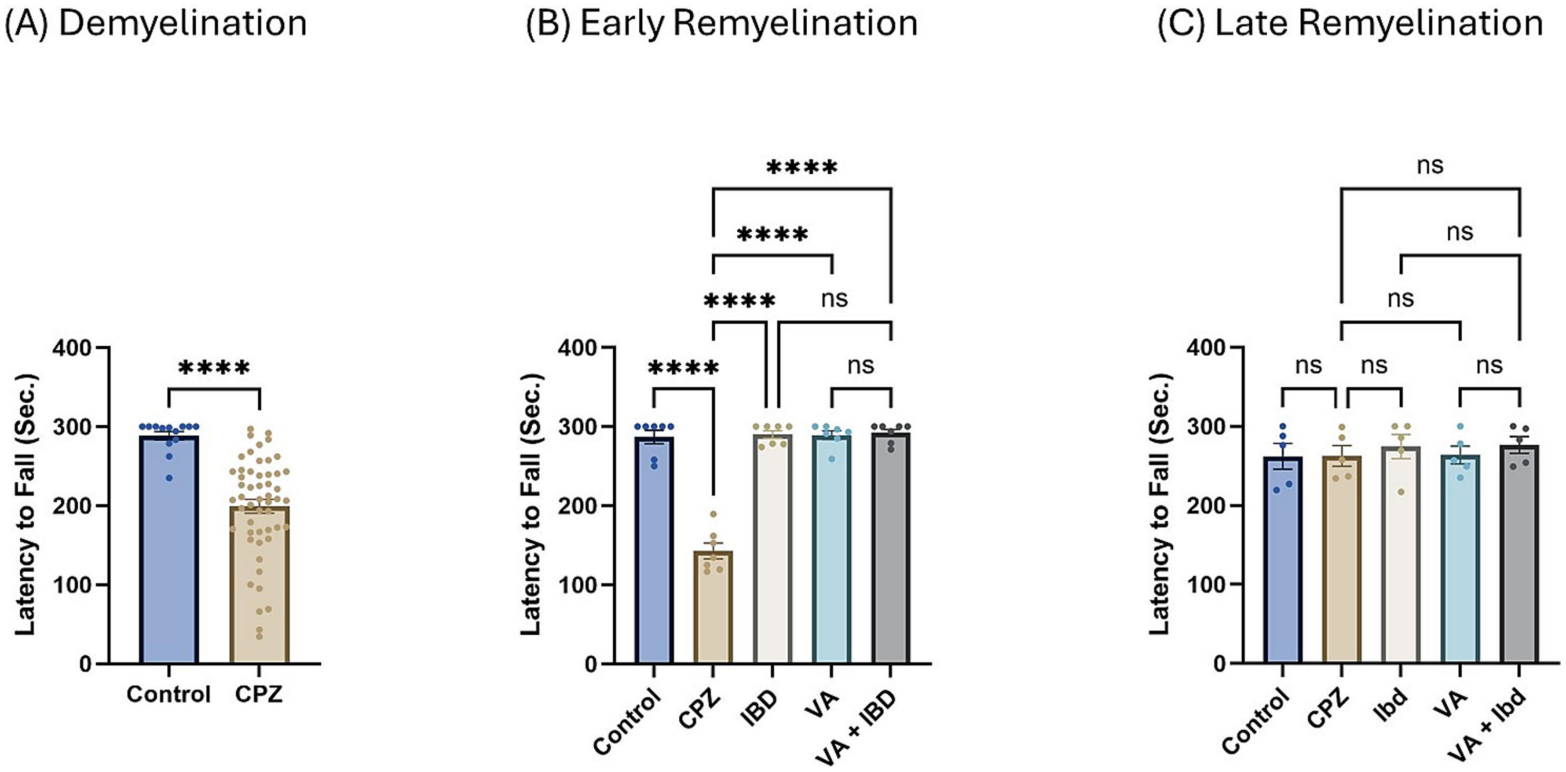
Latency to fall measured using Rotarod test. **(A)** Latency to fall in week 5 of demyelination measured in control and CPZ group. **(B)** Latency to fall of control, CPZ, IBD, VA, and VA + IBD measured in week 7 of early remyelination. **(C)** Latency to fall of control, CPZ, IBD, VA, and VA + IBD measured in week 9 at late remyelination. Data in **(A)** was analyzed by unpaired two-tailed t test. Data in **(B,C)** was analyzed using one-way ANOVA followed by Tukey’s multiple comparisons test. Data was presented as mean ± SEM, and *p* < 0.05 was considered significantly different. ns, non-significant; *p < 0.05, and ****p < 0.0001. Sec., second.

In the rotarod (RR) test, latency to fall was measured in seconds. Latency to fall in week 5 of demyelination showed a significant decrease in the CPZ group compared with the control group (Figure 4A). All treated groups (IBD, VA, and VA + IBD) at week 7 of the early remyelination stage showed a significant increase in latency to fall compared with the CPZ group. Conversely, no significant difference was noted between the combined treatments and the individual treatment groups (Figure 4B). By the end of the late remyelination stage (week 9), no significant difference was observed between the CPZ group and other groups (Figure 4C).

#### 3.1.3 Cognition tests

##### 3.1.3.1 Y-maze spontaneous alternation test

**Figure 5:**
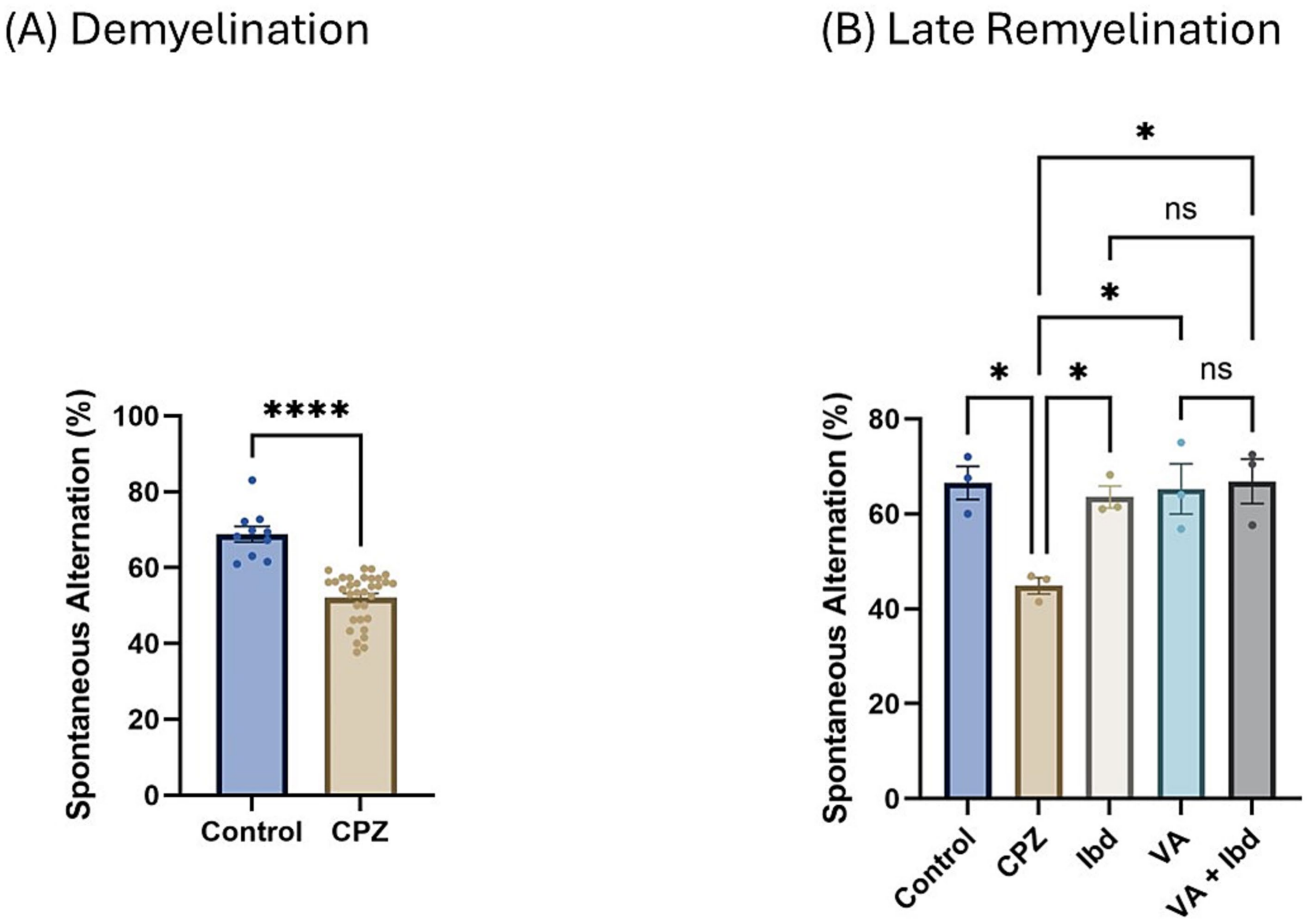
Percentage (%) of spontaneous alteration in Y-maze. **(A)** % of spontaneous alteration was calculated in control and CPZ group at week 5 of demyelination and analyzed by unpaired two-tailed t test. **(B)** % of spontaneous alteration at last week (W 9) of late remyelination shown in control, CPZ, IBD, VA, and VA + IBD. **(B)** Was analyzed using one-way ANOVA followed by Tukey’s multiple comparisons test. Data was presented as mean ± SEM, and *p* < 0.05 was considered significantly different. ns, non-significant; *p < 0.05, and ****p < 0.0001. %, percentage.

At the end of week 5 (demyelination), percent spontaneous alteration was significantly reduced in the CPZ group compared with the control group (Figure 5A). At the end of week 9 (late remyelination), all treated groups besides the control group showed a significant increase in the percentage of spontaneous alteration when analyzed using one-way ANOVA followed by Tukey’s multiple comparisons test compared with the CPZ group. However, no significant difference was shown between the individual treatment groups and the combined treatment group (Figure 5B).

##### 3.1.3.2 Novel object recognition test

In the Novel Object Recognition Test (NORT), two parameters were used to express the results: discrimination index (DI) and preference index (PI) percentage (PI %) (Figures 8, 9 respectively). The DI at the end of the demyelination stage was significantly reduced in the CPZ group compared with the control group (Figure 8A). At the end of the late remyelination stage, the DI was observed with a significant increase in the IBD and VA + IBD groups, but not in the VA group. Further, no significant difference was found between the IBD and VA + IBD groups (Figure 8B). At the end of the demyelination stage, the PI % was calculated and was significantly reduced in the CPZ group compared with the control group (Figure 9A). At the end of the late remyelination stage, a significant increase in PI % was observed in the IBD and VA + IBD groups compared with the CPZ group, which underwent spontaneous remyelination, although such an increase was not observed in the VA-treated group (Figure 9B). Representative track pathways in the NORT using Noldus EthoVision® Video Tracking software at week 5 (demyelination) and week 9 (late remyelination) are shown in Supplementary Figures S5, S6, respectively.

**Figure 6:**
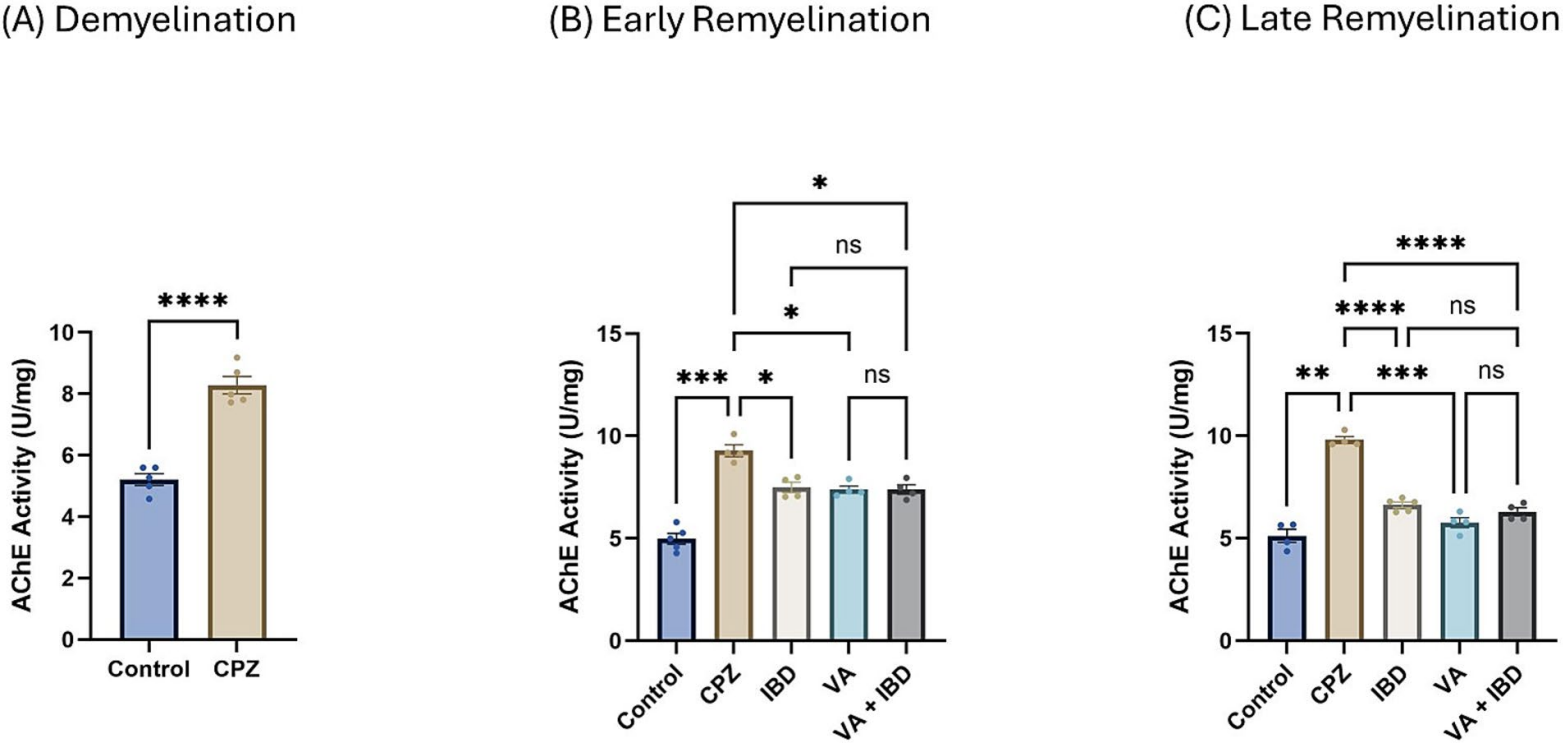
Acetylcholinesterase activity. **(A)** Acetylcholinesterase activity in week 5 of demyelination measured in Control and CPZ group. **(B)** Acetylcholinesterase Activity of Control, CPZ, IBD, VA, and VA + IBD measured in week 7 of early remyelination. **(C)** Acetylcholinesterase Activity of Control, CPZ, IBD, VA, and VA + IBD measured in week 9 at late remyelination. Data in **(A)** was analyzed by unpaired two-tailed t test. Data in **(B,C)** was analyzed using one-way ANOVA followed by Tukey’s multiple comparisons test. Data was presented as mean ± SEM, and *p* < 0.05 was considered significantly different. ns, non-significant; *p < 0.05, **p < 0.01, ***p < 0.001, and ****p < 0.0001. AChE, Acetylcholinesterase; U, unit; mg, milligram.

**Figure 7:**
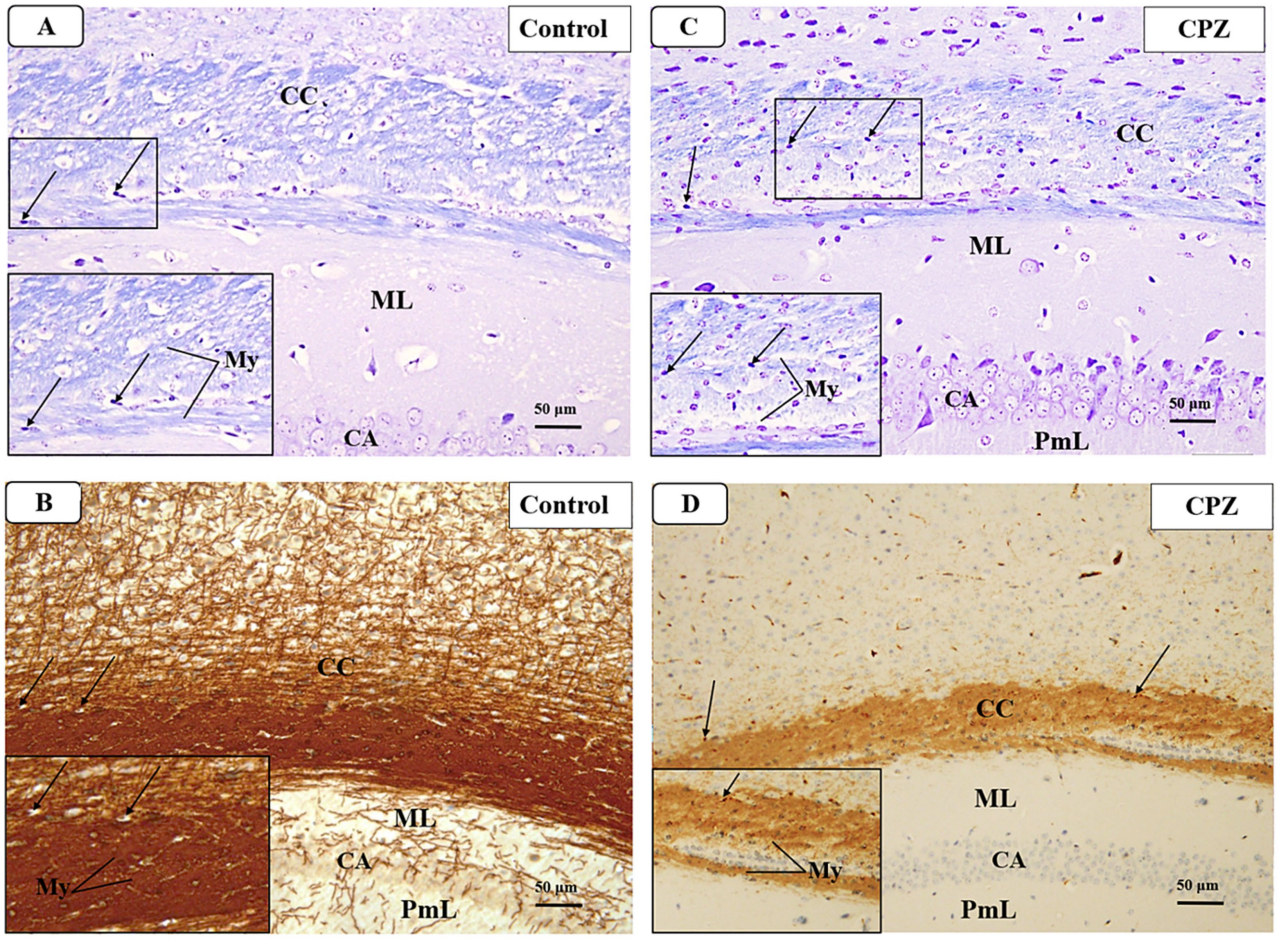
Representative photomicrographs of the corpus callosum (CC) at demyelination. **(A,B)** The Control group showed a typical blue staining intensity and a tightly packed arrangement of myelinated fibers in the corpus callosum (CC). Inset shows regularly dispersed myelinated nerve fibers (My) and oligodendrocytes (arrows). The myelinated nerve fibers are highly defined, darkly pigmented, and are positioned in between the nerve fibers. There is a strong positive reactivity of myelin basic protein in both the myelin fibers (My) and oligodendrocytes (arrows). **(C,D)** The CPZ group exhibited a minor reduction in staining intensity in the previously mentioned areas compared to the control group. The observed findings include a reduction and disorganization in the myelinated nerve fibers (My) and a decrease of oligodendrocytes size (arrows). There is a weak reaction to myelin basic protein of myelin fibers (My) and oligodendrocytes (arrows).

**Figure 8:**
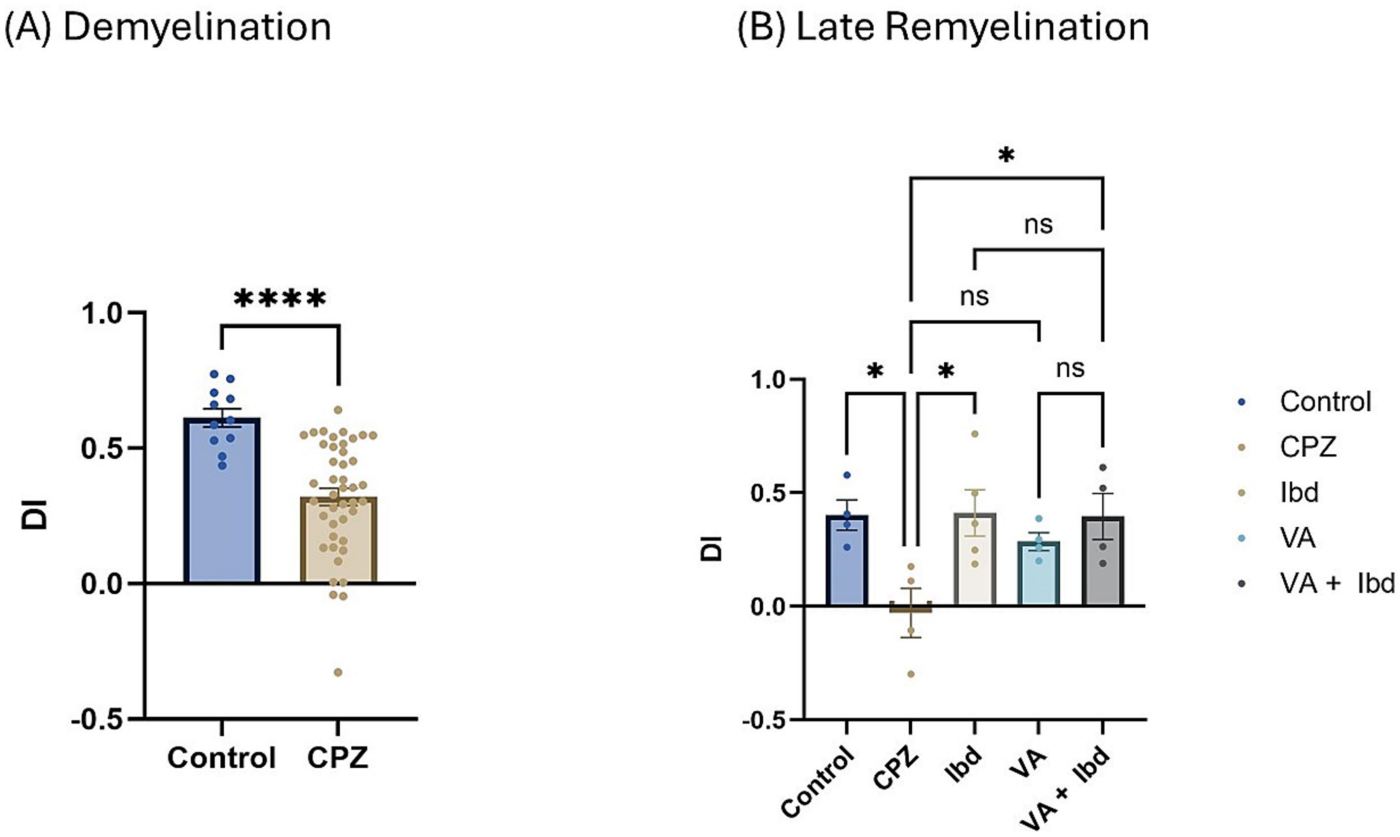
Novel Object Recognition Test (NORT) expressed as Discrimination Index (DI). **(A)** DI measured in week 5 of demyelination in control and CPZ using unpaired two-tailed t test. **(B)** DI measured in week 9 of late remyelination in control, CPZ, IBD, VA, and VA + IBD using one-way ANOVA followed by Tukey’s multiple comparisons test. Data was presented as mean ± SEM, and *p* < 0.05 was considered significantly different. ns, non-significant; *p < 0.05, and ****p < 0.0001. NORT, Novel Object Recognition Test; DI, Discrimination Index.

**Figure 9:**
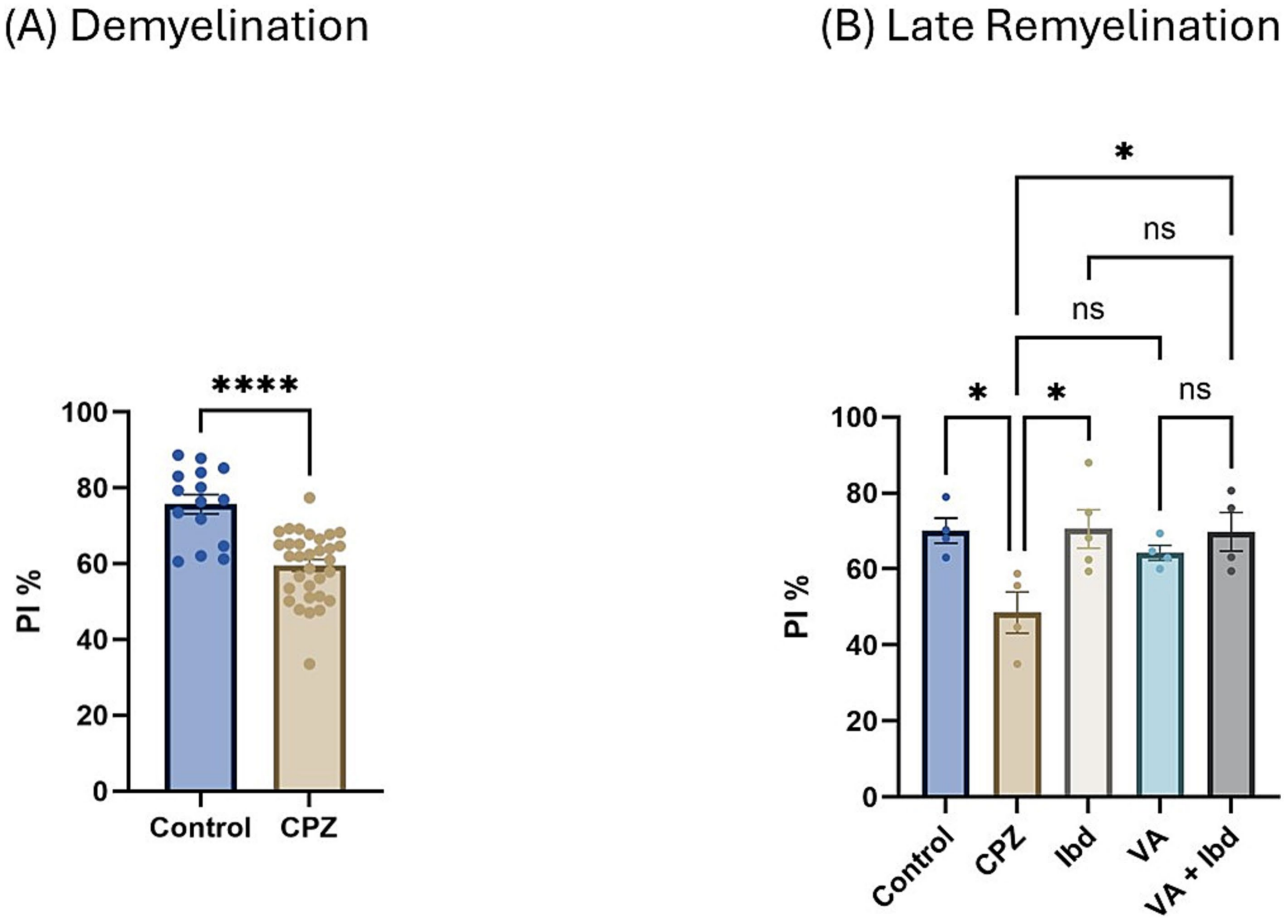
Novel Object Recognition Test (NORT) expressed as Preference Index percentage (PI %). **(A)** PI % measured in week 5 of demyelination in control and CPZ using unpaired two-tailed t test. **(B)** PI % measured in week 9 of late remyelination in control, CPZ, IBD, VA, and VA + IBD using one-way ANOVA followed by Tukey’s multiple comparisons test. Data was presented as mean ± SEM, and *p* < 0.05 was considered significantly different. ns, non-significant; *p < 0.05, and ****p < 0.0001. NORT, Novel Object Recognition Test; PI, Preference Index; %, percentage.

### 3.2 Gene expression

#### 3.2.1 Interleukin-4

**Figure 10:**
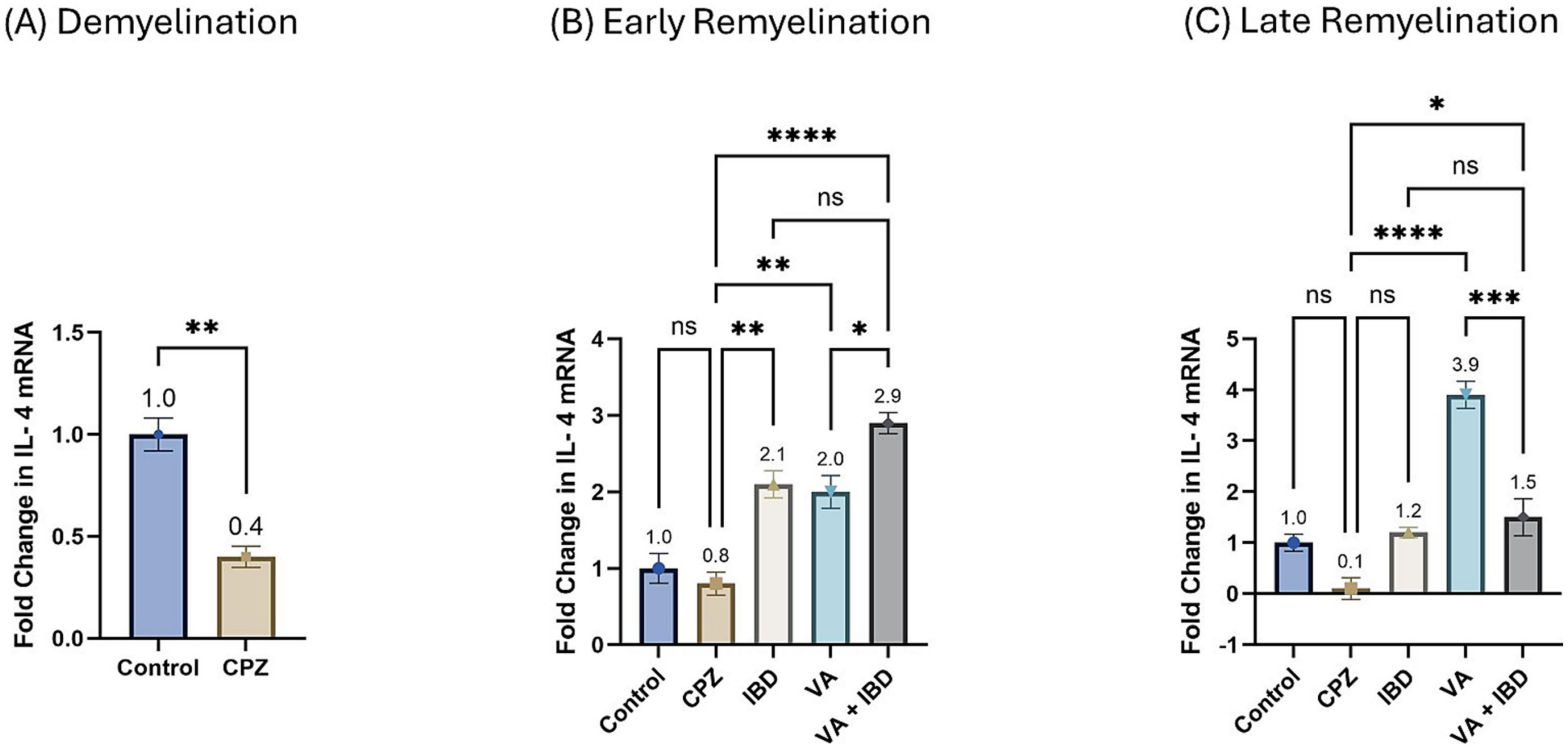
Fold change in Interlukin-4 mRNA. **(A)** Fold change in Il-4 in week 5 of demyelination measured in Control and CPZ group. **(B)** Fold change in Il-4 of Control, CPZ, IBD, VA, and VA + IBD measured in week 7 of early remyelination. **(C)** Fold change in Il-4 of Control, CPZ, IBD, VA, and VA + IBD measured in week 9 at late remyelination. Data in **(A)** was analyzed by unpaired two-tailed t test. Data in **(B,C)** was analyzed using one-way ANOVA followed by Tukey’s multiple comparisons test. Data was presented as mean ± SEM, and *p* < 0.05 was considered significantly different. ns, non-significant; *p < 0.05, **p < 0.01, and ****p < 0.0001.

Interlukin-4 (IL-4) gene expression presented as fold change in mRNA at week 5 during the demyelination phase was found to be decreased significantly in the CPZ group compared with the control group (Figure 10A). At the end of the early remyelination stage (week 7), a significant increase in the fold change in mRNA levels in the treatment groups like IBD, VA, and VA + IBD groups was observed compared with the CPZ group, which underwent spontaneous remyelination. Contrarily, mRNA levels of IL-4 in the Control and CPZ groups were observed to be equivalent. Further, the combined treatment was found with significantly elevated levels of IL-4 mRNA compared with the VA treatment group, but not to the IBD group (Figure 10B). At end of the late remyelination stage (week 9), a significant increase in the mRNA levels of IL-4 was noted in the VA and VA + IBD groups compared with the CPZ group, which underwent spontaneous remyelination. However, no significant change in IL-4 levels was observed in the IBD and control groups compared with the CPZ group. Additionally, no significant change in mRNA of IL-4 was observed between the combined and individual treatment groups (Figure 10C).

#### 3.2.2 Cyclooxygenase-2

**Figure 11:**
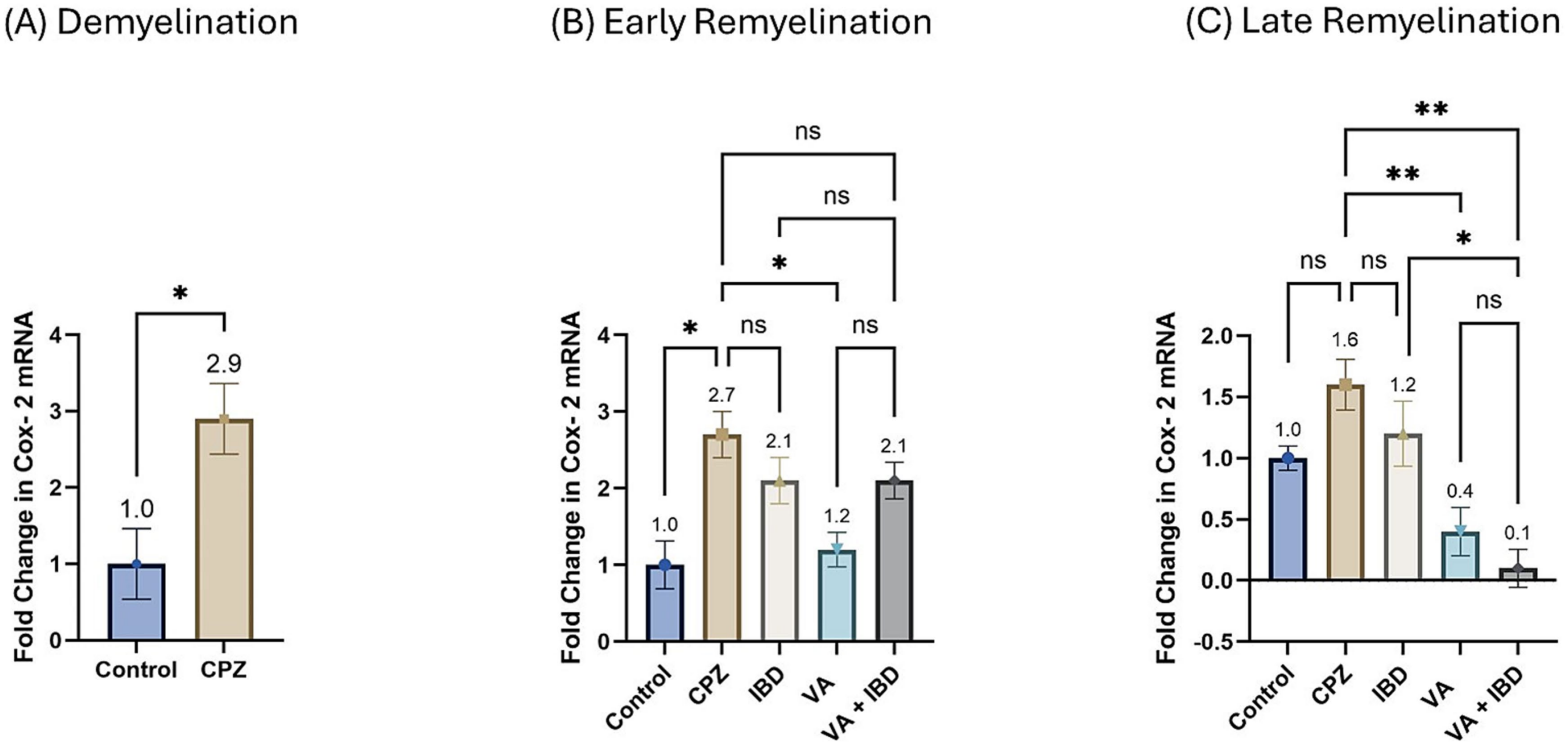
Fold change in Cyclooxygenase-2 (Cox-2) mRNA. **(A)** Fold change in Cox-2 in week 5 of demyelination measured in Control and CPZ group. **(B)** Fold change in Cox-2 of Control, CPZ, IBD, VA, and VA + IBD measured in week 7 of early remyelination. **(C)** Fold change in Cox-2 of Control, CPZ, IBD, VA, and VA + IBD measured in week 9 at late remyelination. Data in **(A)** was analyzed by unpaired two-tailed t test. Data in **(B,C)** was analyzed using one-way ANOVA followed by Tukey’s multiple comparisons test. Data was presented as mean ± SEM, and *p* < 0.05 was considered significantly different. ns, non-significant; *p < 0.05, and **p < 0.01.

The fold change in terms of the mRNA expression of COX-2 during the demyelination stage was found to be significantly increased in the CPZ group compared with the control group (Figure 11A). Two weeks of treatment with different drugs and their combinations resulted in a decrease of COX-2 mRNA levels compared with the CPZ group. Statistical analysis revealed a significant reduction in COX-2 mRNA levels in the VA-treated group compared with the control group. No statistically significant difference was observed between the combined VA + IBD group and the single drug (VA/IBD) treatment group (Figure 11B). Four weeks of treatment (week 9) resulted in a significant decrease of COX-2 mRNA in the VA and VA + IBD groups compared with the CPZ group, which underwent spontaneous remyelination. The IBD treatment did not cause a significant decrease in COX-2, whereas the VA + IBD combination has shown a significant difference in COX-2 levels compared with the with IBD treatment alone but not with VA treatment alone (Figure 11C).

### 3.3 Cholinergic enzyme activity

#### 3.3.1 Acetylcholinesterase activity

In the CPZ-treated group the acetylcholinesterase (AChE) activity was found to be significantly increased compared with the control group at the end of week 5 (Figure 6A). At week 7, all treated groups and the control group have shown a significant decrease in the AChE activity compared with the CPZ group with no significant difference between the combined treatment group and the single compound treatment group (Figure 6B). Similar results were observed at the end of week 9 (late remyelination) (Figure 6C).

### 3.4 Histopathology and immunohistochemistry

#### 3.4.1 Demyelination

At week 5, a microscopic examination of slides stained with Luxol Fast Blue (LFB) from the Control and CPZ groups was conducted. A high concentration of tightly packed myelinated fibers organized in a parallel manner inside the corpus callosum (CC) was seen with blue coloration in the control group. The nuclei of oligodendrocytes were clearly visible, rounded, and darkly stained. They were placed in between the myelinated fibers (Figure 7A). The IHC of Myelin basic protein (MBP) revealed a strong positive reaction in the nerve fibers and the nuclei of oligodendrocytes (Figure 7B). Conversely, the CPZ-treated group exhibited a decrease in the intensity of LFB staining in the CC, where the fibers were disordered and loosely packed. The oligodendrocytes exhibited an uneven form and were small, displaying a weak staining pattern (Figure 7C). Additionally, the MBP IHC analysis revealed weak reactivity in the nerve fibers and the nuclei of oligodendrocytes (Figure 7D).

#### 3.4.2 Early Remyelination

**Figure 12:**
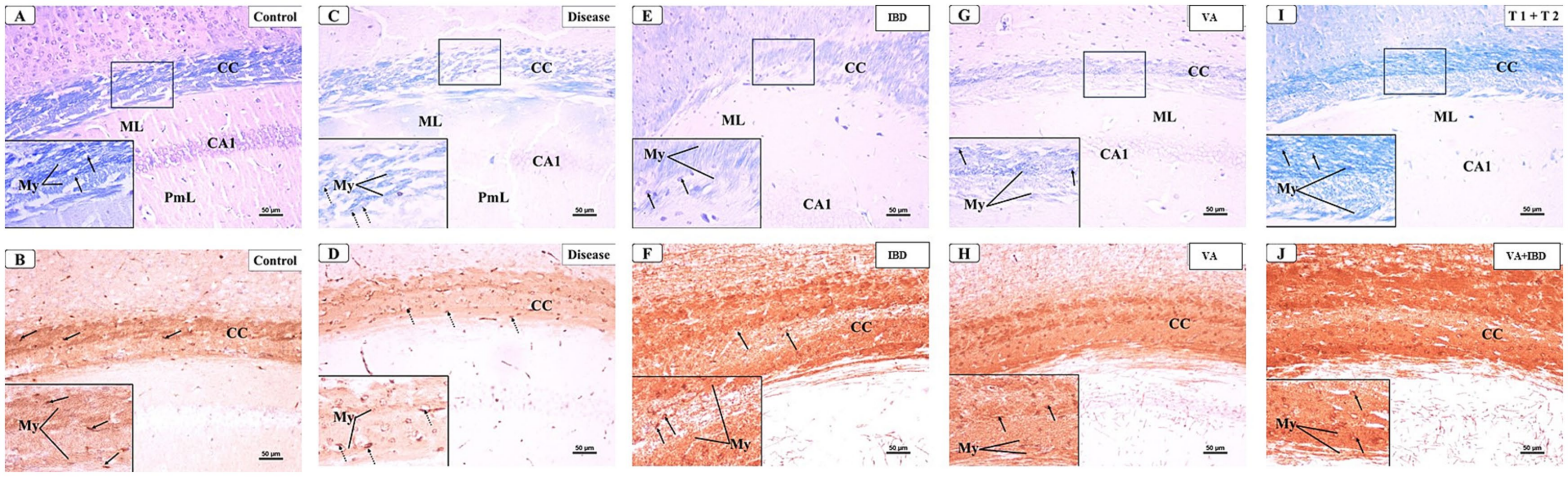
Representative photomicrographs of the corpus callosum (CC) at early remyelination. **(A,B)** Control group showed normal dense blue staining intensity and densely packed, myelinated fibers in the corpus callosum (CC). Inset displayed normally distributed myelinated nerve fibers (My) and oligodendrocytes (arrows) that appear well defined, darkly stained and arranged in between the nerve fibers. Moderate positive myelin basic protein reaction of myelin fibers (My) and oligodendrocytes (arrows). **(C,D)** CPZ group showed marked decrease in staining intensity in the above areas as compared to control. Inset displayed decreased myelinated nerve fibers (My) and less number of oligodendrocytes of small size (doted arrows). Weak myelin basic protein reaction of myelin fibers (My) and oligodendrocytes (arrows). **(E,F)** IBD group showed mild increase in staining intensity in the above areas as compared to the CPZ group. Inset displayed the presence of some demyelinated nerve fibers (My) and oligodendrocytes (arrows). Positive myelin basic protein reaction of myelin fibers (My) and oligodendrocytes (arrows). **(G,H)** VA group showed mild increase in staining intensity in the above areas as compared to the CPZ group. Inset displayed the presence of less demyelinated nerve fibers (My) and oligodendrocytes (arrows) from CC. Moderate myelin basic protein reaction of myelin fibers (My) and oligodendrocytes (arrows). **(I,J)** VA + IBD showed blue staining intensity in the above areas more or less similar to control group. Inset displayed the presence of myelinated nerve fibers (My) and oligodendrocytes (arrows) from CC. Strong myelin basic protein reaction of myelin fibers (My) and oligodendrocytes (arrows). ML: molecular layer; PmL: pleomorphic layer; CA1: first part of cornu ammonis. **(A,C,E,I)** Luxol fast blue stain (LFB) × 200; **(B,D,F,J)** Myelin basic protein (MBP) × 200.

The microscopic examinations of LFB-stained sections were again conducted in week 7. The control group was found to have densely packed, myelinated, and parallel arranged fibers in the CC region, which were stained dark blue. The nuclei of oligodendrocytes appeared well defined, rounded, darkly stained, and arranged between the myelinated fibers (Figure 12A). The IHC of MBP revealed a positive reaction in the nerve fibers and the nuclei of oligodendrocytes (Figure 12B). The CPZ-treated group exhibited marked reduction in the intensity of the LFB stain of the CC, and most fibers appeared disorganized and pale in color. The oligodendrocytes appeared irregular in shape and small with faint staining (Figure 12C). A weak reaction was observed regarding the IHC of MBP in the nerve fibers and the nuclei of oligodendrocytes (Figure 12D). In the IBD-treated group, some CC nerve fibers appeared demyelinated and disorganized with light LFB staining, while the remaining nerve fibers appeared well arranged with partial loss of oligodendrocytes (Figure 12E). The nerve fibers exhibited a moderate positive reaction when IHC was done against MBP (Figure 12F). The CC nerve fibers were found to be partially demyelinated in the VA-treated group. Moreover, some areas exhibited splitting of the nerve fibers. In this group, the oligodendrocytes were found to be preserved and arranged in between the nerve fibers (Figure 12G). The MBP IHC showed that the nerve fibers had mild positive reactions (Figure 12H). In the combined treatment group (IBD + VA), the CC nerve fibers were found with moderate color intensity. Further, the oligodendrocytes appeared preserved and arranged in between the nerve fibers (Figure 12I). The MBP IHC showed that the nerve fibers had strong positive reactions (Figure 12J).

#### 3.4.3 Late remyelination

**Figure 13:**
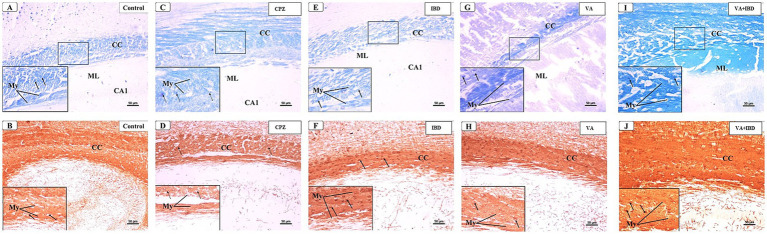
Representative photomicrographs of the corpus callosum (CC) at late remyelination. **(A,B)** Control group showed normal dense blue staining intensity and densely packed, myelinated fibers in the corpus callosum (CC). Inset displayed normally distributed myelinated nerve fibers (My) and oligodendrocytes (arrows) that appear well defined, darkly stained, and arranged in between the nerve fibers. Strong positive myelin basic protein reaction of myelin fibers (My) and oligodendrocytes (arrows). **(C,D)** CPZ group showed marked decrease in staining intensity in the above areas as compared to control. Inset displayed decreased myelinated nerve fibers (My) and less number of oligodendrocytes of small size (doted arrows). Mild myelin basic protein reaction of myelin fibers (My) and oligodendrocytes (doted arrows). **(E,F)** IBD group showed an increase in staining intensity in the above areas as compared to CPZ. Inset displayed the presence of some demyelinated nerve fibers (My) and oligodendrocytes (arrows). Strong myelin basic protein reaction of myelin fibers (My) and oligodendrocytes (arrows). **(G,H)** VA group showed moderate increase in staining intensity in the above areas as compared to the CPZ group. Inset displayed the presence of less demyelinated nerve fibers (My) and oligodendrocytes (arrows) from CC. Moderate myelin basic protein reaction of myelin fibers (My) and oligodendrocytes (arrows). **(I,J)** VA + IBD group showed strong blue staining intensity in the above areas. Inset displayed the presence of myelinated nerve fibers (My) and oligodendrocytes (arrows) from CC. Strong myelin basic protein reaction of myelin fibers (My) and oligodendrocytes (arrows). ML, molecular layer; PmL, pleomorphic layer; CA1, first part of cornu ammonis. **(A,C,E,I)** Luxol fast blue stain (LFB) × 200; **(B,D,F,J)** Myelin basic protein (MBP) × 200.

The microscopic examination of LFB and IHC of MBP stained sections from the control group (Figures 13A,B) showed similar findings as that of the control group of the early remyelination phase. The LFB staining of the CC in the CPZ-treated group likewise in the early remyelination phase showed a significant decrease in intensity, with most fibers appearing fragmented and bright in color. Moreover, the oligodendrocytes appeared irregularly shaped, relatively small, and faintly stained (Figure 13C). The MBP IHC showed mild positive reactions in the nerve fibers and the nuclei of oligodendrocytes (Figure 13D). The IBD-treated group showed few demyelinated and disorganized CC fibers, while the remaining nerve fibers appeared well arranged but with partial loss of oligodendrocytes (Figure 13E). The MBP IHC also exhibited that the nerve fibers had strong positive reactions compared with the CPZ group (Figure 13F). The VA-treated group affirmed that the CC nerve fibers had minimal demyelination. The oligodendrocytes appeared preserved and arranged in between the nerve fibers (Figure 13G). The MBP IHC exhibited the nerve fibers and Ols with strong positive reactions compared with the CPZ group (Figure 13H). In the VA + IBD-treated group, the CC nerve fibers appeared more or less similar to the control group with restored color intensity. Moreover, the oligodendrocytes appeared preserved and arranged in between the nerve fibers (Figure 13I). The MBP IHC, similar to the control group, showed that the nerve fibers and oligodendrocytes had strong positive reactions compared with the CPZ group (Figure 13J).

## 4 Discussion

This study was aimed to investigate the therapeutic effect(s) of the natural product VA, and the anti-inflammatory drug IBD individually and in combination on CPZ mouse-model of MS.

To the best of our knowledge, this is the first study to demonstrate the therapeutic potential of IBD and VA on an acute MS model, making it a suitable candidate for preclinical investigation of MS treatment. Also, the possibility of posing a synergistic effect was tested as well in this study. The novelty of the therapeutic approach was achieved by reaching the optimal acute demyelination prior to the administration of the treatments.

There are only a few studies investigated the effect of VA on the mice cuprizone model. One of them that was carried out by Safwat et al. (2024) has investigated the prophylactic effect of VA on the CPZ model. They have not investigated the therapeutic effects of VA, hence the remyelination was not addressed. Also, they examined the demyelination in the peripheral nervous system (PNS) in contrast to our study where we examined the brain. Moreover, the parameters investigated were different as well as the model rodent (Safwat et al., 2024). The other study exploring the impact of VA on CPZ model was published in a conference abstract by our group in 2022 investigated only anxiety behaviors, not any other parameter (Alderbi et al., 2022). Therefore, this is the first study which examines the neurotherapeutic impact of VA on different aspects of remyelination in CPZ model of MS.

Several behavioral deficits, molecular alterations, and histological changes occur when CPZ is given to mice for 5 weeks as reported previously (Zhan et al., 2020). Behavioral changes, such as decreased mobility, weakness of GS, balance ability, and cognitive impairments, have been noted due to CPZ intoxication (Aldhahri et al., 2022; Han et al., 2020). CPZ also causes oligodendrocyte loss leading to demyelination (Han et al., 2020; Zirngibl et al., 2022).

In our study, we observed that 5-week intoxication with CPZ resulted in demyelination as revealed by histological studies, as confirmed by a significant decrease in motor ability, a decrease in cognition, an increase in a cholinergic enzyme activity, and alteration in relevant gene expression compared with the control group upon CPZ intoxication (week 5). These findings support the evidence from previous studies that CPZ causes locomotion and coordination deficits, and cognitive impairment (Al-Otaibi et al., 2022; Aldhahri et al., 2022; Zhang et al., 2022) determined by the mentioned test. The histopathological findings also are directly in line with previous findings that CPZ causes oligodendrocyte loss leading to demyelination proven by relevant histopathological tests (Han et al., 2020; Zirngibl et al., 2022). Together, the results of the tests conducted in the 5^th^ week of CPZ intoxication have proven the succession of the MS model.

Two weeks following the 5-week CPZ intoxication phase, at the seventh week of the study, biochemical alterations were detected, indicating the existence and ongoing process of remyelination. Hence, this may be the best window of opportunity to research the molecular processes behind remyelination, identifying critical times for early therapeutic intervention (Palavra et al., 2022). Therefore, in the current study, week 7 was chosen for conducting most of the behavioral, histological, and molecular assessments.

Interestingly, at the early remyelination phase, it was found that VA exerts a promising improvement as noted during locomotion tests, balance tests, molecular and biochemical tests, and histopathological evaluation. The hanging time, the normalized force, and the TDM that were all decreased in the demyelination phase have revealed a significant improvement in the VA-treated group (Figures 1B, 2B, 3B). Similarly, the IBD group showed a significant improvement in outcomes for the same parameters when compared with the group that did not receive any treatment. Moreover, the group that received a combination of VA and IBD exhibited an enhancement in all the parameters tested (Figures 1B, 2B, 3B respectively). Furthermore, the latency to fall measured in the rotarod test was significantly enhanced in the VA-treated group and the IBD group compared with the untreated group (Figure 4B). While Interlukin-4 (Il-4) has a pleotropic role in inflammation (Gärtner et al., 2023; Urcelay et al., 2005), it was significantly decreased in CPZ group compared to control in demyelination (Figure 10A), suggesting that Il-4 in this model played an anti-inflammatory role with regard to the other supporting results which mentioned the strong anti-inflammatory effects of Il-4, and its role in the clinical illness observed in Il-4 defective animals (Urcelay et al., 2005). In the early remyelination, a significant increase in the gene expression of Il-4 (Figure 10B) was seen indicating that the treatment with VA ameliorates the demyelination at molecular level in agreement with the literature (Ingole et al., 2021; Sharma et al., 2020). Furthermore, the expression of Cyclooxygenase-2 (Cox-2), known for its role in pro-inflammation (Turini and DuBois, 2002), was significantly decreased after treatment with VA as (Figure 11B). Another important finding is that the AChE activity which has been linked to the pathology of MS (Di Bari et al., 2016), was significantly decreased in mice treated with VA, IBD, and VA + IBD (Figure 6B) confirming the enhancement of remyelination. It has been suggested that inhibiting AChE activities in the brain could play a role in the management of neurodegenerative disorders, particularly those associated with cholinergic dysfunction (Ijomone and Obi, 2013; Omotoso et al., 2018).

The histopathological evaluation at the early remyelination phase revealed that the VA group and IBD group demonstrated only partial demyelination in the CC nerve fibers with few instances of splitting in the myelin fibers (Figures 12E,G,I), whereas the CPZ-treated group showed a significant decrease in the intensity of LFB staining in the CC, with most fibers appearing disordered and light in color (Figure 12C). The oligodendrocytes in the CPZ group exhibited an uneven form and were very tiny, displaying a weak staining, while those in the slides prepared from the treated groups were well preserved and organized (Figures 12C,E,G,I). Additionally, the IHC results of MBP showed nerve fibers with mild positive reactions in the VA group, IBD, and combined group (Figures 12F,H,J), while the CPZ group (spontaneous remyelination or untreated) displayed a weak reaction (Figure 12D). The histopathological and immunohistochemical findings of the study are in line with the other findings that VA treatment and IBD treatment demonstrated improvement at the microscopic level on the CPZ model.

Nevertheless, most of the locomotion and coordination tests demonstrated no significant difference in the treated groups when compared with the untreated group at late remyelination, suggesting that spontaneous remyelination occurred in the untreated group.

In reviewing the literature, it was demonstrated that four to 7 weeks after cuprizone withdraw, even after remyelination is complete, some behavioral abnormalities like impaired adaptive motor learning remains (Zirngibl et al., 2022). Furthermore, the affected brain area involved in cognition and memory which hippocampus has shown longer spontaneous remyelination periods which might be extended to a couple of weeks after CPZ withdraw (Cui et al., 2018). Hence, conducting cognitive behaviors in week 9 of the study was more feasible than week 7 of the study.

We found that parameters to determine cognition, such as spontaneous alteration (Figure 5A), the DI (Figure 8A), and the PI (Figure 9A), were significantly decreased in the CPZ group compared with the control group. These results are in accordance with earlier studies (Al-Otaibi et al., 2022; Aldhahri et al., 2022; Zhang et al., 2022). In the current study, we observed that spatial memory deficits in the CPZ group improved with VA treatment and IBD treatment (Figure 5B), as assessed by the Y-maze test. Contrary to expectations, VA showed no significant improvement in the NORT at week 9 of the study although there was an increase in the DI (Figure 8B) and PI % (Figure 9B). A possible explanation for this might be the demand for a longer treatment regimen and/or a higher dose of VA. Among most of the tests conducted, no significant difference was seen between the individually treated groups with VA and IBD and their combination, suggesting that each works independently in a different pathway.

VA promotes the upregulation of Akt/GSK-3β/Nrf2 signaling pathways, therefore enhancing HO-1 expression. This specialized method enables vanillic acid to serve as a powerful antioxidant (Amin et al., 2017; El-Sheikh et al., 2023). Vanillic acid inhibits the receptor-interacting protein (RIP)-2/caspase-1 pathway and nitric oxide (NO) generation, leading to an anti-inflammatory response in mouse peritoneal macrophage cells (Kim et al., 2011). Vanillic acid has also been reported to mitigate oxidative damage via Adenosine Monophosphate-Activated Protein Kinase Signaling Pathway (Ma et al., 2019). IBD is a phosphodiesterase inhibitor acts by inhibiting the cleavage of cyclic adenosine monophosphate (cAMP) (Goodman et al., 2016). While this is a different pathway than VAs’, hence, the absence of synergism between IBD and VA can be explained by their independent pathways.

In summary, the results of this study indicate the novelty of vanillic acid therapeutic impact on different behavioral, molecular, biochemical, and histological tests conducted on a cuprizone mouse-model of multiple sclerosis. These results were shown on locomotion and coordination behavioral tests conducted at early remyelination phase of the study, whereas the impact of vanillic acid on spatial memory was seen on late remyelination phase. Also, the improvement of acetylcholinesterase inhibition, the increase in the expression of Il-4, the reduction in Cox-2 expression, and the evaluation of the histopathology and immunohistochemistry, altogether have supported the idea that vanillic acid ameliorates the pathological manifestation of the model starting from early remyelination. Ibudilast has exerted a therapeutic effect on most of the tests as expected, nevertheless, no consistent significant difference was seen in the combined treatment of vanillic acid and Ibudilast.

Further radiological, biochemical, and molecular investigations are recommended to explain the underlying mechanisms by which vanillic acid improved the behavioral deficits of the cuprizone model. Moreover, it is recommended to investigate the feasibility of recruiting vanillic acid as a potential treatment to Multiple sclerosis. VA up to 50 mg/kg I.P. is known to be safe (Hajhashemi et al., 2022), and yet far from the I.P. LD50 which is 2,691 mg/kg in mice according to the safety data sheet of Cayman Chemicals. Moreover, the window of the treatment can be extended to 6 weeks of treatment as the 6^th^ week is overlapped between demyelination and remyelination as addressed by Al-Otaibi et al. (2022).

The main limitation of the CPZ model in this study is that there was no distinct manifestation of demyelination during the *in vivo* phase; the only reliable method to confirm demyelination is through histopathological tests which require rodents sacrificing. The decline seen on the affected mice can assist in evaluating the demyelination yet insufficient when applied independently. This can be solved by establishing a protocol that involves Magnetic resonance imaging (MRI), for instance, to evaluate and detect demyelination in vivo. Different MRI techniques such as Diffusion tensor imaging (DTI) and Quantitative susceptibility mapping (QSM) can be used to assess the extent of the demyelination and the remyelination that occur during the study (Wang et al., 2019). Also, performing quantitative analysis of optical density of immunohistochemistry results might support the findings of this study.

Future research could investigate blood tests and brain tissue enzyme-linked immunosorbent assay (ELISA) of certain cytokines and proteins involved in demyelination, as well as exploring additional genes through molecular tests to confirm the initial findings of this study. Furthermore, electron microscopy could be implemented to evaluate cytopathology during the course of study, and involves oligodendrocytes, microglia, and astrocytes assessment besides the neurons.

## Data Availability

The raw data supporting the conclusions of this article will be made available by the authors upon request.
