# Supplementary material for: Neurotherapeutic impact of vanillic acid and ibudilast on the cuprizone model of multiple sclerosis
Source: Front Mol Neurosci. 2025 Jan 10;17:1503396. doi: 10.3389/fnmol.2024.1503396 (PMC11760597; doi:10.3389/fnmol.2024.1503396)
Supplement: Supplementary file 1 [file Table_1.docx]

Supplementary Material

# Supplementary Figures


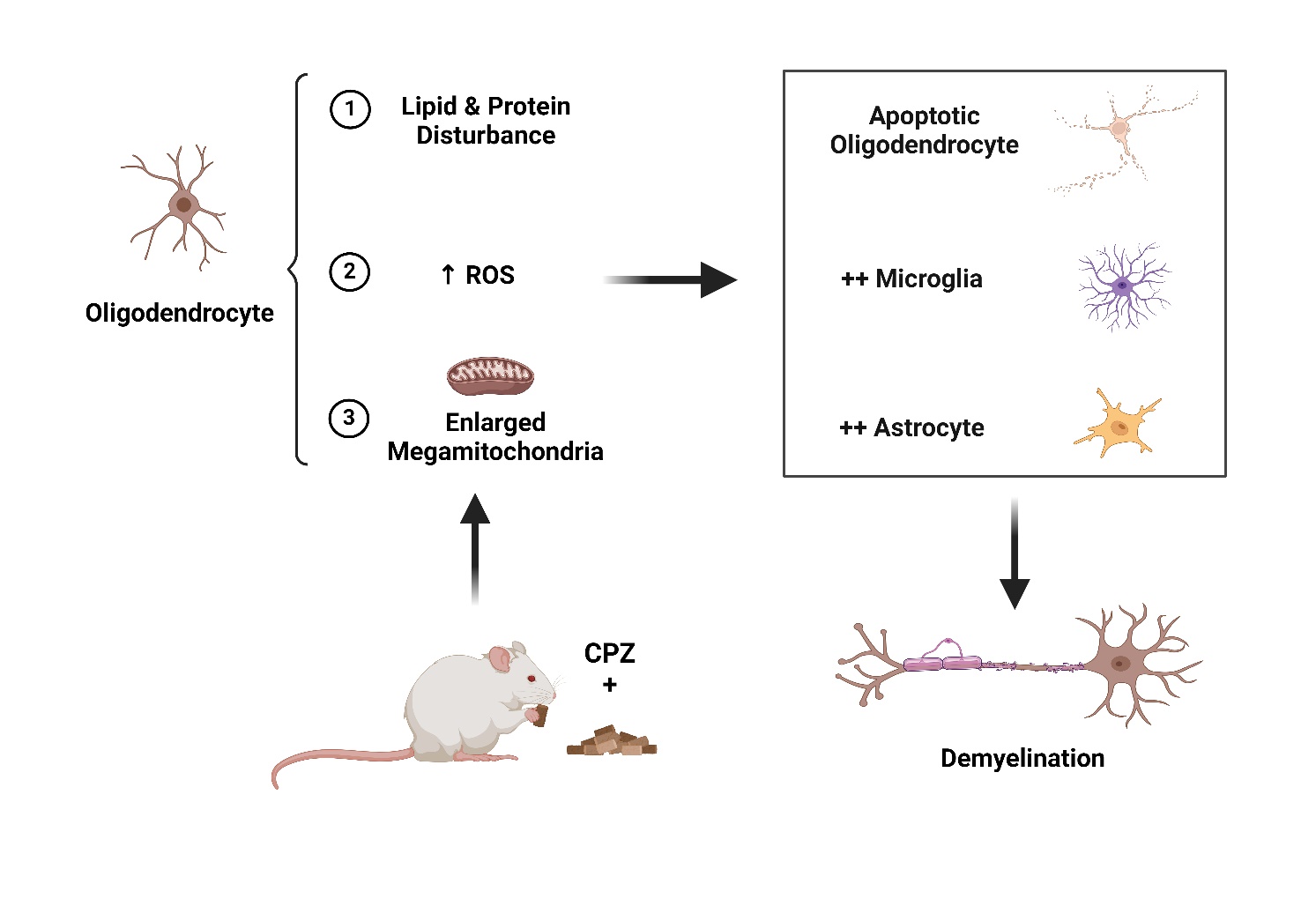


**Supplementary Figure 1.** Cuprizone mechanism of demyelination. Created with Biorender.com

**
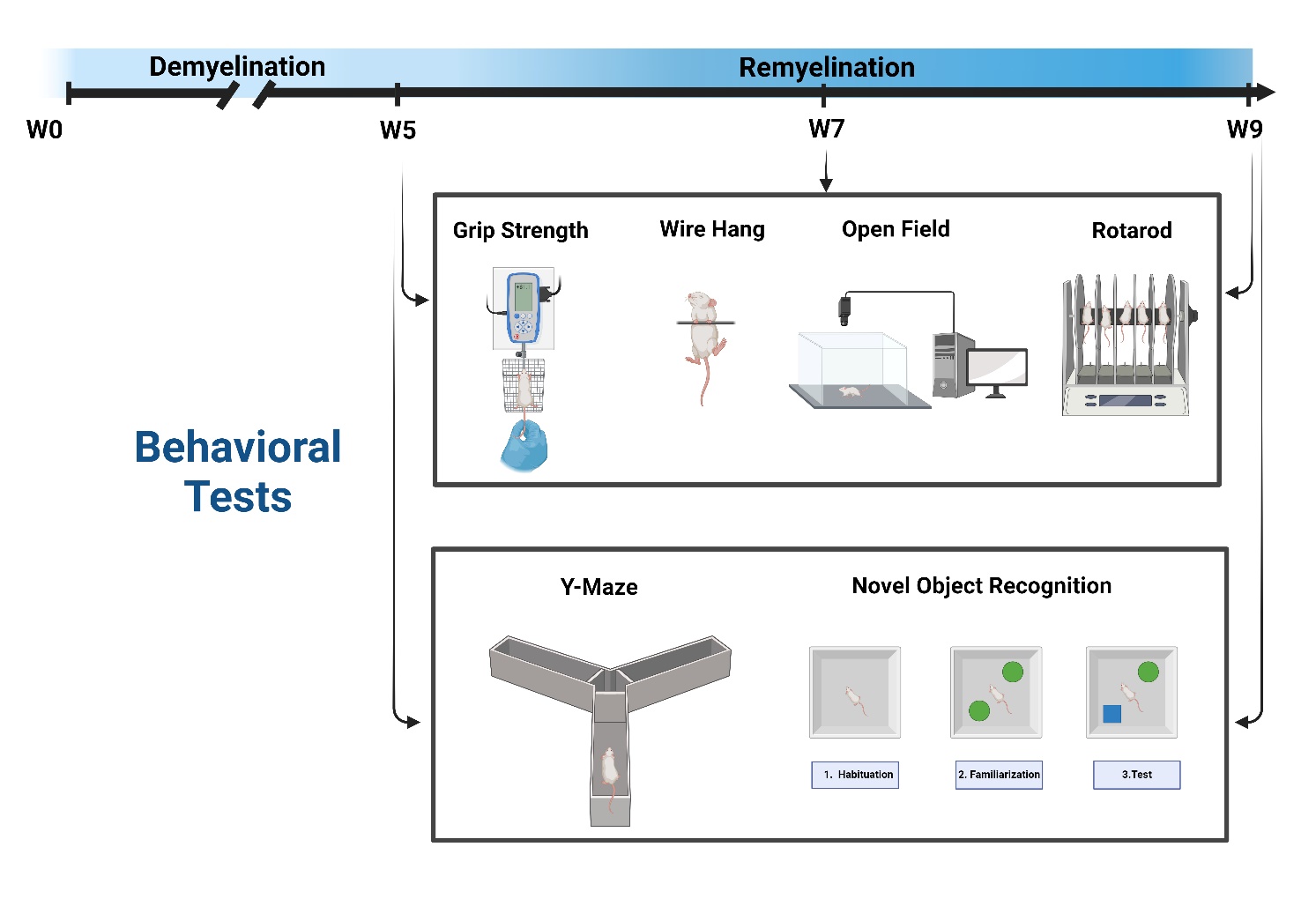
**

**Supplementary Figure 2.** Experimental Design (behavioral tests). Created with Biorender.com


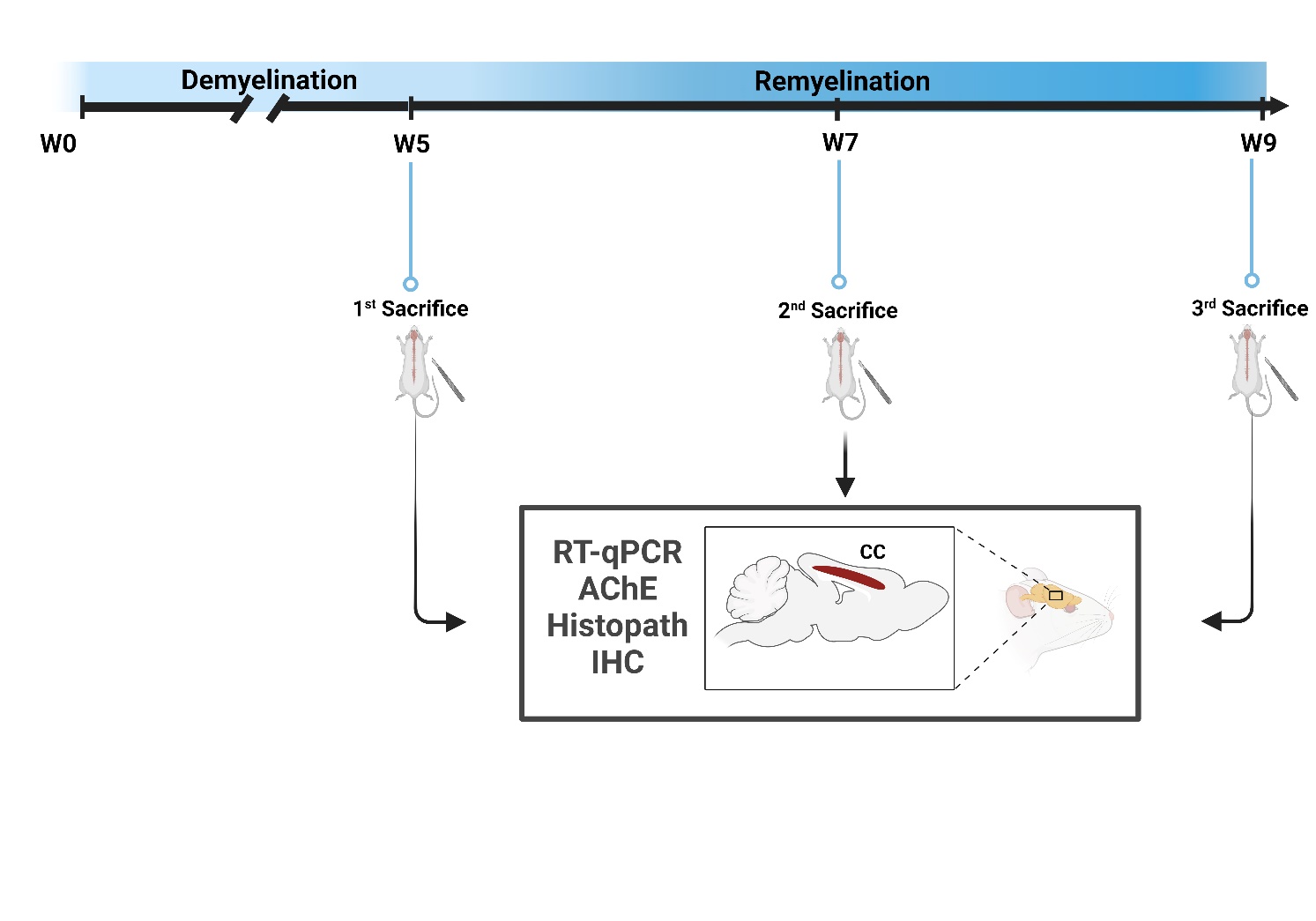
**Supplementary Figure 3.** Experimental Design (molecular, biochemical, and histopathological tests). RT-qPCR, quantitative reverse transcription real-time polymerase chain reaction; AChE, acetylcholinesterase activity; Histopath, histopathology; IHC, immunohistochemistry. Created with Biorender.com


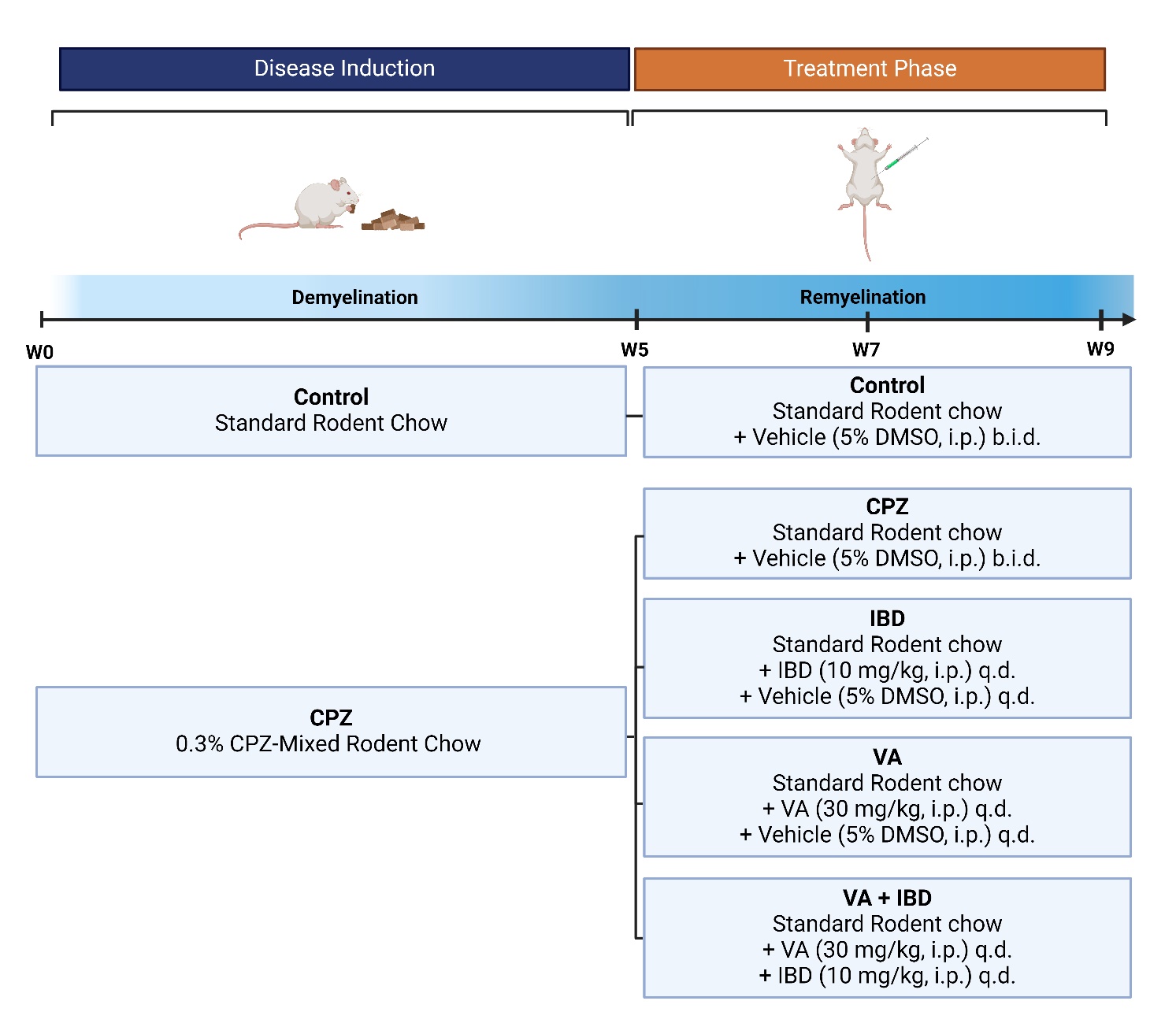


**Supplementary Figure 4.** Mice Grouping. DMSO, Dimethylsulfoxide; CPZ, Cuprizone; IBD, Ibudilast; VA, Vanillic acid; i.p., intraperitoneal; q.d., once daily, b.i.d.; twice daily. Created with Biorender.com


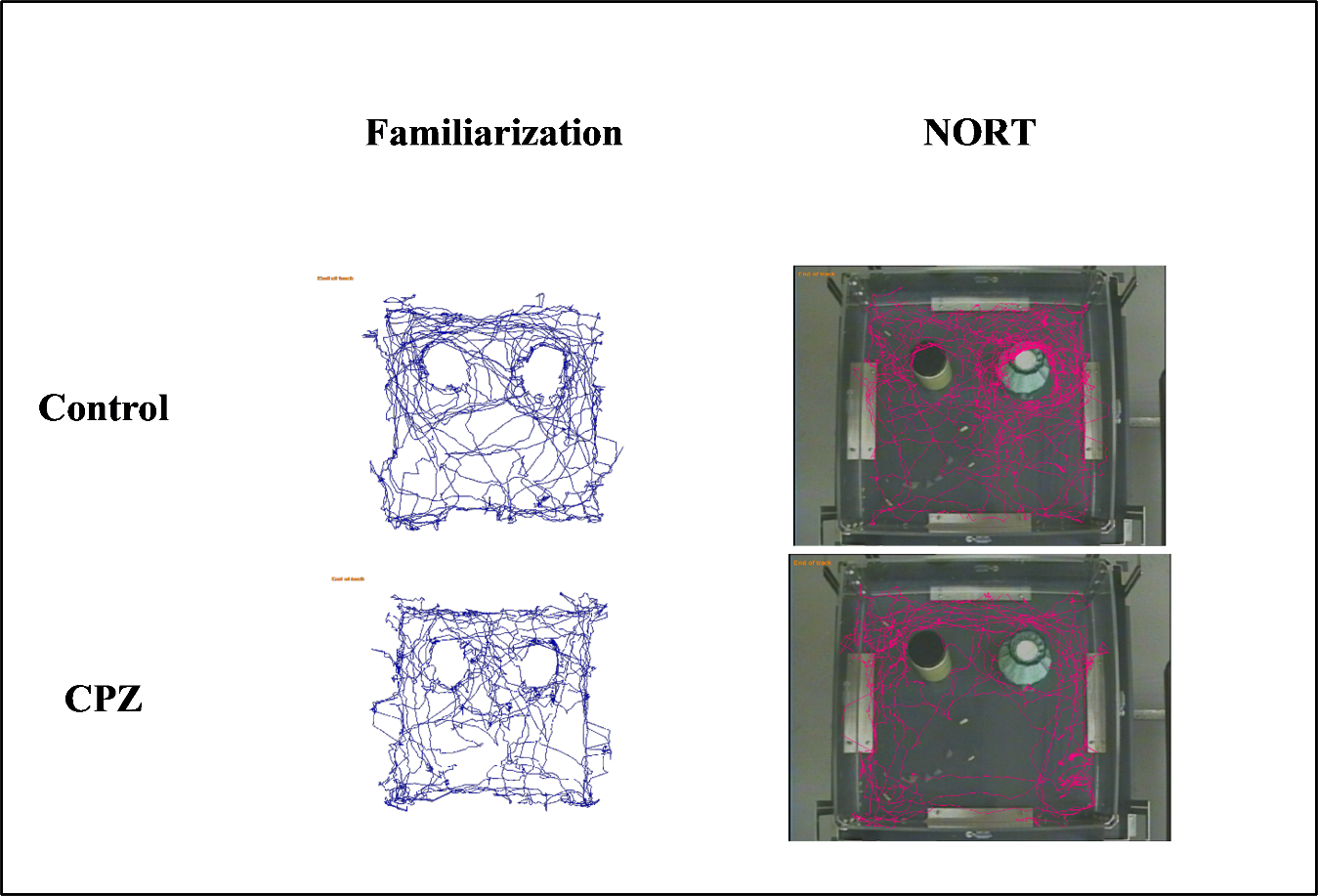


**Supplementary Figure 5.** Representative Track Pathway in Novel Object Recognition Test (NORT) at week 5 (Demyelination). The novel object is the one on the right side of the pictures.


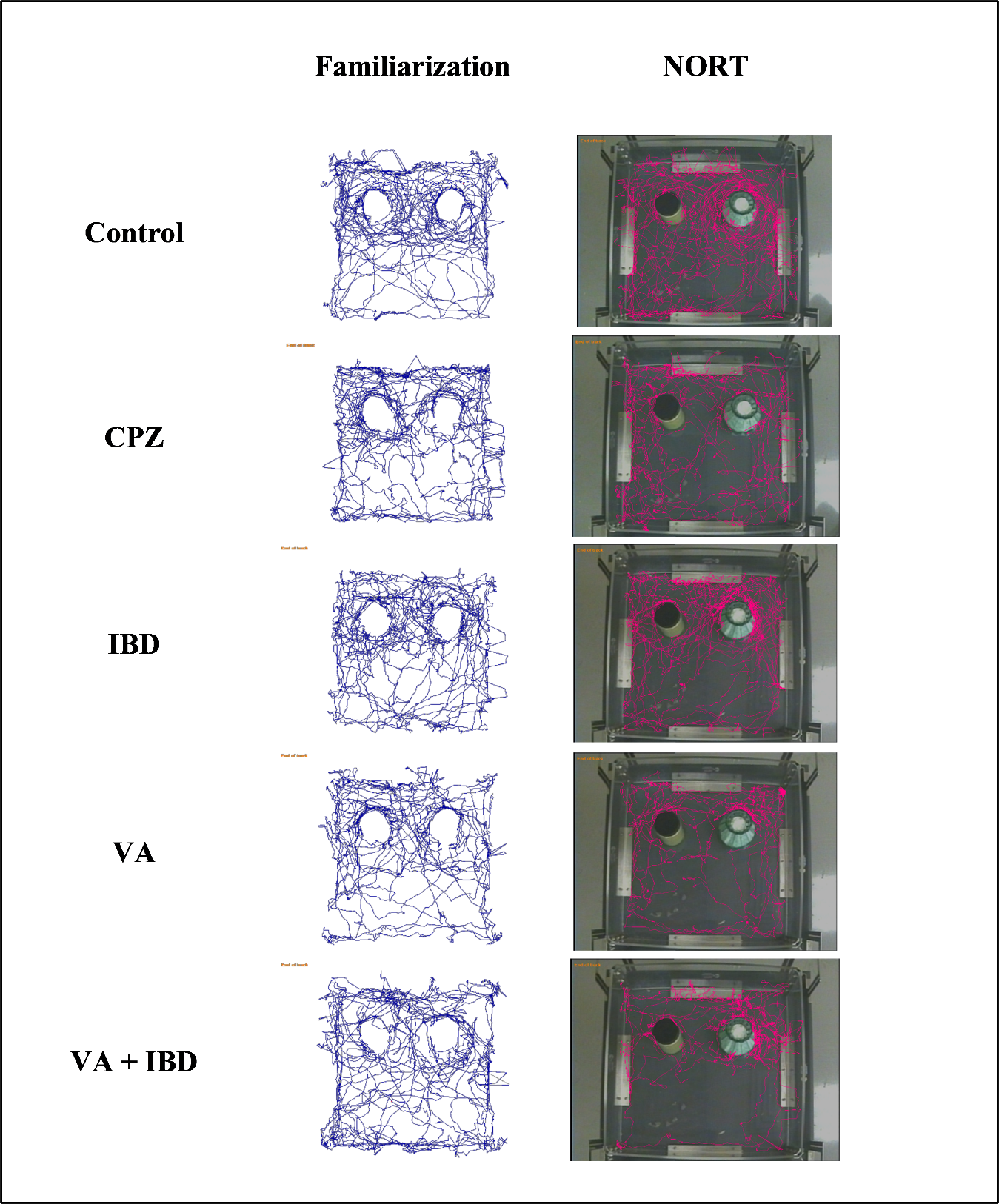


**Supplementary Figure 6.** Representative Track Pathway in Novel Object Recognition Test (NORT) at week 9 (Late Remyelination). The novel object
